# Amazonian trees under long-term drought exposure are more resilient to El Niño extreme

**DOI:** 10.1038/s43247-026-03887-0

**Published:** 2026-09-14

**Authors:** Lion R. Martius, Pablo Sanchez-Martinez, Vanessa Negrão-Rodrigues, Paulo Bittencourt, Wout Cherlet, Wouter van den Broeck, Zane Cooper, Louise Terryn, Caterina Buranelli, Calil Torres-Amaral, Ross Deans, Oliver Binks, Mateus C. Silva, Anthony Barbosa da Silva, Renan Domingues Pacheco, Grazielle Sales Teodoro, João Athaydes Silva Junior, Kim Calders, Mathias Disney, Sassan Saatchi, Edward T. A. Mitchard, Rafael S. Oliveira, Lucy Rowland, Antonio Carlos Lola da Costa, Maurizio Mencuccini, Patrick Meir

**Affiliations:** 1 School of GeoSciences, University of Edinburgh, Edinburgh, UK; 2 Universidade Federal Rural da Amazônia, Belém, Pará Brazil; 3 Museu Paraense Emílio Goeldi, Belém, Pará Brazil; 4 School of Earth and Environmental Sciences, Cardiff University, Cardiff, UK; 5 Department of Geography, Faculty of Environment, Science and Economy, University of Exeter, Exeter, UK; 6 Q-ForestLab – Laboratory of Quantitative Forest Ecosystem Science, Department of Environment, Ghent University, Ghent, Belgium; 7 Instituto de Geociências, Universidade Federal do Pará, Belém, Pará Brazil; 8 CREAF, Campus UAB, Cerdanyola del, Vallés, Spain; 9 Instituto de Ciências Biológicas, Universidade Federal do Pará, Belém, Pará Brazil; 10 Department of Geography, University College London, London, UK; 11 NERC National Centre for Earth Observation (NCEO), London, UK; 12 Jet Propulsion Laboratory, California Institute of Technology, Pasadena, CA USA; 13 Space Intelligence Ltd., Edinburgh, UK; 14 Department of Plant Biology, Institute of Biology, CP 6109, University of Campinas – UNICAMP, Campinas, Brazil; 15 ICREA, Barcelona, Spain

**Keywords:** Tropical ecology, Plant physiology, Phenology

## Abstract

The rising frequency and intensity of extreme droughts across the Amazon raises uncertainty about the capacity of tropical forests to adjust to future climate. Here, we combined the severe 2023 El Niño drought with a multi-decadal throughfall-exclusion experiment to compare eco-physiological resilience between pre-exposed drought-primed trees (PT) and untreated controls (CT). During El Niño, PTs reduced transpiration by 25%, while CTs declined by up to 54% relative to pre-drought levels. Post-drought, PTs fully recovered transpiration, whereas CTs recovered only to 77.5%, showing legacy effects and pronounced leaf area loss. These differences were reinforced by long-term structural changes (i.e., mortality) in the TFE, which reduced water competition during extreme drought. Tree structural data revealed a significantly smaller crown-to-stem size ratio in PTs, reducing sensitivity to evaporative demand and underpinning their enhanced drought resistance and resilience. These findings reveal that physiological resilience to extreme events can emerge from long-term drought exposure through structural adjustments at both ecosystem and individual tree scale.

## Abstract (web summary)

Long-term drought exposure enhances Amazon tree resilience to extreme drought through structural adjustments at ecosystem and tree levels, which reduces transpiration declines and enables full recovery, based on El Niño observations and a multi‑decadal throughfall‑exclusion experiment.

## Introduction

Amazon rainforests exchange large quantities of carbon dioxide and latent heat with the atmosphere, and contribute significantly to the stability of the Earth’s climate system^1–3^. However, Amazonia’s functional stability is facing substantial risks due to global warming and deforestation^4–9^. Recent decades have experienced an increase in severe drought events across the Amazon basin, associated with sea surface temperature (SST) anomalies linked to the El Niño–Southern Oscillation (ENSO) in the tropical Pacific and warming SST in the Tropical North Atlantic (TNA), with the majority of severe droughts in the Amazon being related to ENSO^10–12^. While ENSO and warming of the SST in the TNA are natural climate phenomena, their intensity, duration and frequency have increased^11,13^. In the last two decades, four droughts (TNA: 2005, 2010; ENSO: 2015/16, 2023/24) were ranked as “once-in-a-century” events, each exceeding the previous in magnitude^14,15^. These climate anomalies were characterised by prolonged reductions in rainfall and soil moisture, alongside elevated vapour pressure deficit, air temperatures, and solar radiation across the basin^15^. The impacts on the tropical land surface have been linked with net CO_2_ emissions to the atmosphere, driven by a weakening terrestrial carbon sink, associated with amplified physiological stress in trees (i.e., reduced photosynthesis and elevated tree mortality) and a higher fire incidence^1,16–18^. The recent ENSO 2023/24 triggered one of the most severe droughts on record in the Amazon^10,15^, likely exposing tropical trees to immense hydraulic stress diminishing forest function^19–22^.

Thus, quantifying physiological resistance, defined as the ability to maintain physiological function during drought and resilience, that is the capacity of trees to recover key functions like transpiration or carbon assimilation, is essential to assess drought impacts and legacy effects^23–25^. Although transpiration is not a direct proxy for growth, its maintenance during drought and recovery after rewetting reflect the integrity of hydraulic function, and thereby can capture drought legacies linked to carbon assimilation, potential repair costs, and structural adjustments such as reduced leaf area^24,26^. However, while short-term drought responses of tropical trees are relatively well studied, they are unlikely to fully predict long-term trajectories of resistance and resilience under future extremes^27^. Bridging short and long timescales is increasingly urgent given climate predictions of prolonged dry seasons and more frequent extreme drought events^11,14,27,28^.

From a biogeographical perspective, studies across rainfall gradients in Amazonia show communities shifting towards a higher abundance of drought-affiliated species with inherently more drought-tolerant traits, highlighting species turnover and trait plasticity as key responses^29,30^. However, hydraulic trait plasticity and adjustment to drought on the scale of individual adult trees seems to be limited^31,32^. Instead, individuals may undergo structural adjustments to modify architecture and optimise water use under stress in response to long-term drought exposure, potentially reflecting phenotypic structural plasticity^33,34^. Such structural adjustments may enable trees to modulate leaf water potential by balancing leaf area, sapwood conducting area, and height relative to atmospheric and soil moisture conditions, as has been observed within species along climatic gradients^33,35,36^. Reductions in transpiring surface area and volume can lower boundary layer and canopy conductance and decrease evaporative demand, enhancing water-use efficiency under recurring stress^34,37^. Such long-term modifications complement and shape short-term responses such as stomatal regulation and leaf shedding, as strategies for trees to control water status during drought^26,38^. Trees with large, hydraulically demanding crowns are therefore expected to rely more on tight stomatal regulation than smaller-crowned trees during acute drought. The degree of water potential regulation, or stringency, describes how strongly trees constrain declines in plant water potential in response to increasing atmospheric evaporative demand and soil moisture limitation^39^.

Insights into how Amazon rainforest trees could respond to multi-decadal drought exposure have been gained from a long-term throughfall-exclusion experiment (TFE, 50% exclusion for >20 years)^26,27,40,41^. The effect of the TFE treatment was to simulate the recurrence of an El Niño-type soil drought by exacerbating dry-season water limitation, increasing the length and intensity of each dry season (Methods). The first year of treatment in 2002 triggered rapid physiological and structural responses, including sharp declines in sap flow, stomatal conductance, and leaf area during the dry season^26^. Leaf area never fully recovered and became a lasting legacy of drought exposure^40^. Significantly elevated tree mortality occurred during the first 15 years of TFE treatment, leading to substantial biomass loss^27^. After seven additional years, totalling 23 years of TFE, the forest was shown to be no longer experiencing elevated tree mortality. This suggested ecohydrological stabilisation via ecosystem-level structural change, with the surviving trees now accessing sufficient water without hydraulic stress^41^. How trees within a forest that has been subjected to long-term drought respond physiologically to acute drought events remains unknown. The El Niño drought of 2023 offered a unique natural experiment to assess how trees exposed to multi-decadal soil drought respond to climatic extremes - conditions expected to become more frequent^14,28^. One potential scenario could be that these trees were ‘primed’ and therefore better able to withstand the 2023/24 El Niño than trees in the untreated control forest. ‘Priming’ refers to the process by which recurrent past stress (priming stimulus; here TFE) modifies responses to future stress (trigger stimulus; here ENSO drought), acting on the phenotype such that long-term adjustments enhance physiological resistance and resilience^25,42^. Alternatively, the extreme conditions during the dry season of 2023 may have exceeded the trees’ fundamental tolerance of drought, leaving them very sensitive to intensifying climate change^28^.

We investigated the physiological responses of Amazonian rainforest trees to the El Niño 2023/24 drought at a uniquely long-term drought experiment in the eastern Amazon^27^. We quantified drought resistance (*Rt*), the ability of a tree to limit the reduction in transpiration during drought, and post-drought resilience (*Rs*), the capacity of trees to reach or exceed pre-drought transpiration levels^23,43^. We measured plant water use, water status regulation stringency^39^, water potential, temporal changes in leaf area index (LAI) and tree and forest structure derived from terrestrial laser scanning (TLS). We compared the physiological performance of PTs subjected to >20 years of recurrent soil drought within the TFE with that of CTs, which have experienced natural rainfall. We tested the following hypotheses: 1) The trees ‘primed’ (PTs) under the long-term recurrent soil drought treatment display higher physiological resistance and resilience to an acute extreme drought event compared to untreated control trees (CTs). 2) The PTs exhibit a lower demand for water during the drought, allowing these trees to endure the extended water limitation imposed by the acute drought compared to CTs. 3) The PTs show hydraulic co-ordination across physiology and tree structural adjustments that together minimise internal water stress during drought.

We show that trees pre-exposed to multi-decadal experimental drought were significantly more resistant and resilient to the El Niño extreme than untreated controls with respect to tree-level transpiration, and achieved full recovery after the El Niño-enhanced dry season. CTs failed to recover transpiration rates and displayed significant post-drought legacy effects alongside pronounced leaf area loss. These differences were reinforced by long-term structural changes (i.e., mortality) in the TFE, which reduced water competition during extreme drought. Significantly smaller relative crown size in PTs compared to CTs resulted in a lower sensitivity to evaporative demand and underpinned their greater resistance to the El Niño drought. These findings reveal that resilience to acute drought can emerge from long-term drought exposure through structural adjustments at the individual tree scale, with emergent feedback at ecosystem scale.

## Results

### Extreme climate in the Amazon during the El Niño 2023

The drought of the El Niño in 2023 was marked by the lowest dry-season precipitation levels since recording started in 1982 at our study site in Caxiuanã National Forest Reserve, in the eastern Amazon. The site receives an average of 227 ± 105 mm of rainfall during the dry season (determined by precipitation ≤ 100 mm month^−1^, August–November) calculated over the period of 1982–2022, but in 2023 it only received 82 mm during those 4 months, while the dry season was also extended by an additional month, resulting in a strongly intensified and extended dry season (Supplementary Fig. 1). In September and October 2023, the forest was in a state of an exceptional meteorological soil drought quantified by the Standardized Precipitation Index accumulated over three months (SPI-3 <−2.0). In addition, seasonal atmospheric conditions during the 2023 El Niño dry season were exceptionally severe compared to the 22-year baseline (2001–2022). Daily maximum above-canopy air temperatures averaged 33.5 ± 0.82 °C during the study period, corresponding to the 98.5th percentile of the historical distribution (2001–2022 mean: 31.5 ± 1.2 °C). Daily maximum vapour pressure deficit (VPD) reached extreme levels with a mean of 2.39 ± 0.39 kPa, ranking at the 91^st^ percentile of the historical data (2001–2022 mean: 1.92 ± 0.39 kPa). Daily minimum relative humidity was severely reduced to 53.8 ± 5.8%, and was placed at the 21st percentile of the historical distribution (2001–2022 mean: 58.4 ± 6.4%). The most extreme atmospheric conditions in the 2001–2023 site climate record all occurred during the 2023 dry season. On 31 October 2023, the site experienced its highest recorded VPD (3.6 kPa), air temperature (36.1 °C), and lowest recorded relative humidity (38.5%) across the full observational time series (Supplementary Fig. 1). Our historical climate data were collected during the past decades of climatic shifts, meaning that our analysis is conservative regarding the detection of extreme events. However, these results underline that this Amazonian forest was exposed to unusually extreme atmospheric drought conditions during the El Niño drought in 2023.

### Drought-primed trees are more resistant during El Niño

**Fig. 1:**
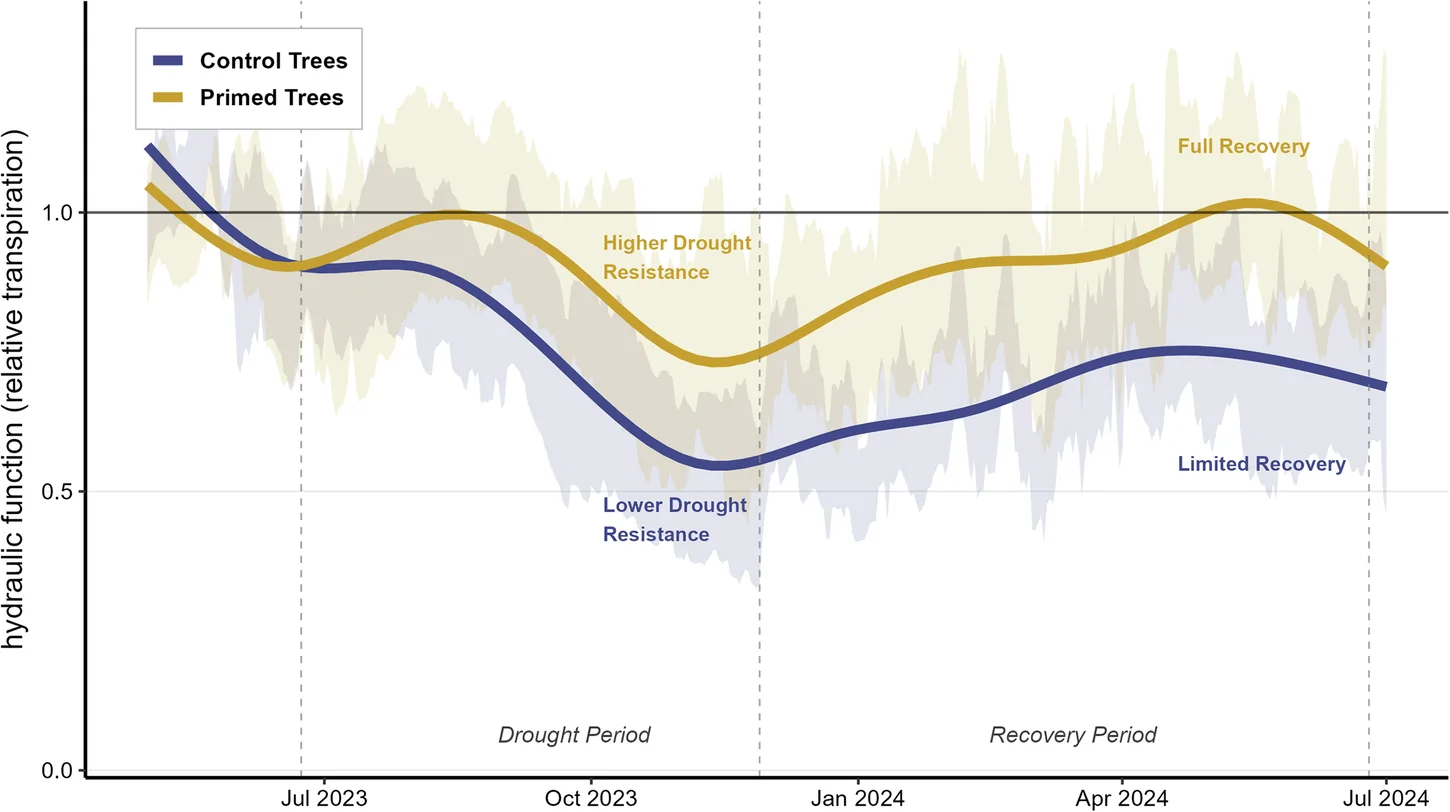
Drought-primed trees are more resilient to the El Niño drought in 2023. Hydraulic function, as the ratio of tree-level transpiration [kg day^−*1*^] during drought (drought resistance) and post-drought (resilience) periods relative to the pre-drought baseline (6 weeks prior to drought) of tropical trees in the eastern Amazon. This study compared trees from a control plot (deep blue) with drought-primed trees from a multi-decadal throughfall-exclusion experiment (goldenrod) during and after the 2023 El Niño drought. The depicted lines exhibit plot-level GAM-fitted trajectories, while the shaded areas represent the 95% confidence intervals centred around the daily mean of each plot. Vertical dashed lines delineate the drought onset (23 June 2023) and cessation (28 November 2023). Measured in 40 individuals (20 per plot, see Methods).

The 2023 El Niño drought triggered substantial declines in drought resistance (*Rt*) across all trees, quantified as the ratio of transpiration [kg day^−1^] during drought (Dr) relative to pre-drought (PreDr) baseline levels. We applied a linear mixed model with *Rt* as the response and individual tree identity as a random intercept. Fixed effects included drought duration (days), plot treatment (TFE vs control), crown size (log-transformed crown volume) and their interaction. Primed trees (PTs) and control trees (CTs) showed a similar initial pre-drought baseline (*t* = −0.17, *p* = 0.9), but diverged significantly over time between treatments (*t* = 13.7, *p* < 0.001, Supplementary Table 1, 2). The CTs showed a steeper reduction in their hydraulic transport capacity with a rate of daily decline in *Rt* of −0.0036 day⁻¹ [95% CI = −0.0038, −0.0034], while PTs demonstrated more drought tolerance with a substantially slower decline of −0.0017 day⁻¹ [95% CI = −0.0022, −0.0016] (Fig. 1). At the peak of the drought, on the last day before the wet-season rainfall resumed, PTs displayed a significantly higher mean resilience of 0.75 [95% CI = 0.64, 0.86] compared to CTs with a mean resilience of 0.46 [95% CI: 0.44, 0.54]), computed from estimated marginal means of the linear mixed model (Fig. 1). Crown size contributed significantly to temporal drought resistance dynamics (*t* = −11.7, *p* < 0.001), increasing explained fixed-effect variance (from ${R}_{m}^{2}\,$= *0.16* to *0.20*, ${R}_{c}^{2}\,$= *0.63*). Thus, independent of treatment, trees with larger crown volumes exhibited a more pronounced loss of drought resistance over time. This suggests that trees with large crowns are disproportionally disadvantaged during long periods of drought with high evaporative demand. When soil water availability (to 4 m depth) per unit tree biomass [mm/MgC] was explicitly included in an alternative model, model performance was slightly increased (${R}_{m}^{2}\,$= *0.22*, ${R}_{c}^{2}\,$= *0.65*), with higher water availability associated with higher overall drought resistance (*t* = 9.3, *p* < 0.001). The temporal treatment effect was no longer significant (*t* = − 1.37, *p* = 0.17), indicating that treatment-specific differences in drought resistance trajectories are largely explained by differences in soil water availability per unit biomass. Notably, the effects of crown size on resistance dynamics remained highly significant (*t* = −12.5, *p* < 0.001, Supplementary Table 3). This demonstrates that drought resistance dynamics were jointly governed by soil water availability per unit biomass and crown size-dependent evaporative demand.

**Fig. 2:**
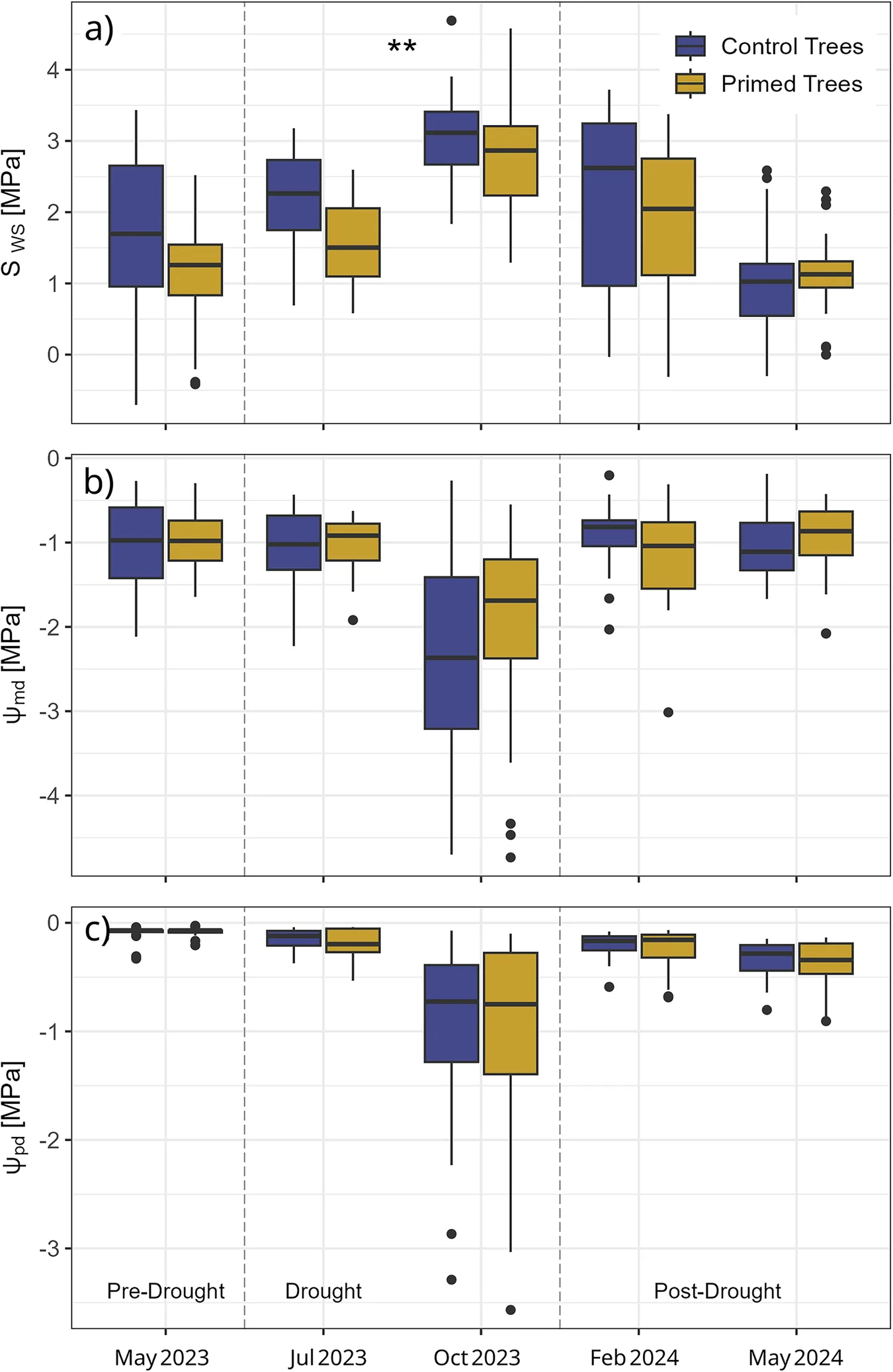
Primed trees show lower levels of water status regulation during El Niño. **a** Plant water status stringency (S^ws^ [MPa]) during five sampling campaigns pre-, during and post- El Niño drought 2023/24. Data are for drought-primed trees (PT) from a long-term throughfall-exclusion experiment and respective control Trees (CT). The *S*^ws^ metric quantifies the departure of observed *Ψ*_md_ from the predictions of a null model representing both thermodynamic forces (i.e., soil and atmospheric drought) when no physiological regulation occurred (Methods Equation 5). **b** Minimum leaf water potentials were measured at midday between 11:45 and 14:00 and **c** pre-dawn leaf water potentials between 04:00 and 06:00 during the same five sampling campaigns in May (pre-), July and October (drought) in 2023, and February and May (post-drought) in 2024. For each variable, we applied two linear mixed models. One over the pre-drought and drought period, and one over the post-drought period, including plot treatment and an interaction with campaign as fixed effects, and tree ID as a random effect. The variables above were measured in the same 40 individuals (20 in the control and 20 in the TFE plot; deep blue = control trees, goldenrod = primed trees). The asterisks mark the significance of the *p* value at 99% level (*p* < 0.01 **) as outputted from the corresponding linear mixed model, related to the plot variable. The absence of asterisks marks the non-significance of plot differences. Our results show that during the acute drought event, PTs regulated their water status significantly less than control trees (*t* = −2.7, *p* = 0.009).

We furthermore assessed how well trees constrained declines in plant water potential in response to the El Niño. We quantified this regulation using a stringency metric, defined as the deviation of observed water potential from a null-reference representing the expected environmental forcing if no regulation by the plants occurred - the higher the stringency, the higher the regulation of water potential (Methods, Equation 5). As the El Niño drought progressed, both CTs and PTs displayed more strict regulation of water potential compared to PreDr. We applied a linear mixed model with plot treatment (TFE or control) and field campaigns as time exposed to drought as fixed effects, with tree ID included as a random effect (${R}_{m}^{2}\,$= *0.42*, ${R}_{c}^{2}\,$= *0.52*). All model terms were significant (Supplementary Table 4). The results showed that during the acute drought event, PTs regulated their water status significantly less by up to −0.46 MPa [95 % CI = −0.8, −0.14] (Fig. 2a), compared to CTs, despite no significant differences in either minimum or pre-dawn leaf water potentials between CTs and PTs throughout the study period (Fig. 2b, c).

### Post-drought resilience and recovery

The moment of return of rainfall initiated the recovery phase. We quantified the post-drought resilience (*Rs*) for each individual tree, as the ratio of transpiration [kg day^−1^] post-drought (PostDr) relative to pre-drought (PreDr) baseline levels. We applied a linear mixed model over the PostDr period, with *Rs* as the response variable, and the same fixed and random effects as the resistance analysis (${R}_{m}^{2}\,$= *0.14*, ${R}_{c}^{2}\,$= *0.73*, Fig. 1). The effect of crown volume was not significant on the post-drought recovery dynamics, and was thus excluded from the model. The PostDr resilience analysis confirmed that PTs demonstrated superior recovery baselines, beginning post-drought recovery from significantly higher hydraulic performance baseline levels due to higher drought resistance (*t* = 2.9, *p* = 0.007), due to the lower loss in relative transpiration during the drought period. The PTs fully recovered transpiration back to PreDr levels after 143 days within the wet-season by end of April 2024. Thus, PTs demonstrated full resilience to the El Niño extreme with no signs of lasting drought legacies in tree transpiration following the extreme drought. In CTs on the other hand, persistent legacy effects were evident. CTs displayed only partial recovery within our observation period, reaching only 77.5% of PreDr levels (Fig. 1, Supplementary Table 1). When soil water availability per unit biomass was explicitly controlled for, the treatment effect remained significant (*t* = 3.2, *p* < 0.001). While soil water availability had a positive effect on recovery (*t* = 5.8, *p* < 0.001), its inclusion only modestly increased the variance explained (${R}_{m}^{2}$ = 0.144 compared with 0.14 in models without soil water). Notably, CTs displayed substantial hysteresis. They failed to recover transpiration to pre-drought levels despite soil water availability per unit biomass returning to baseline, demonstrating a persistent drought legacy effect on function. During the PostDr wet-season, water status regulation was increasingly less strict as the length of time spent exposed to returning rainfall increased, with no differences between plots (${R}_{m}^{2}\,$= *0.17*, ${R}_{c}^{2}\,$= *0.19*, Fig. 2, Supplementary Table 4). There were no significant differences in PostDr water status between treatments.

### Structural differences and long-term drought

**Fig. 3:**
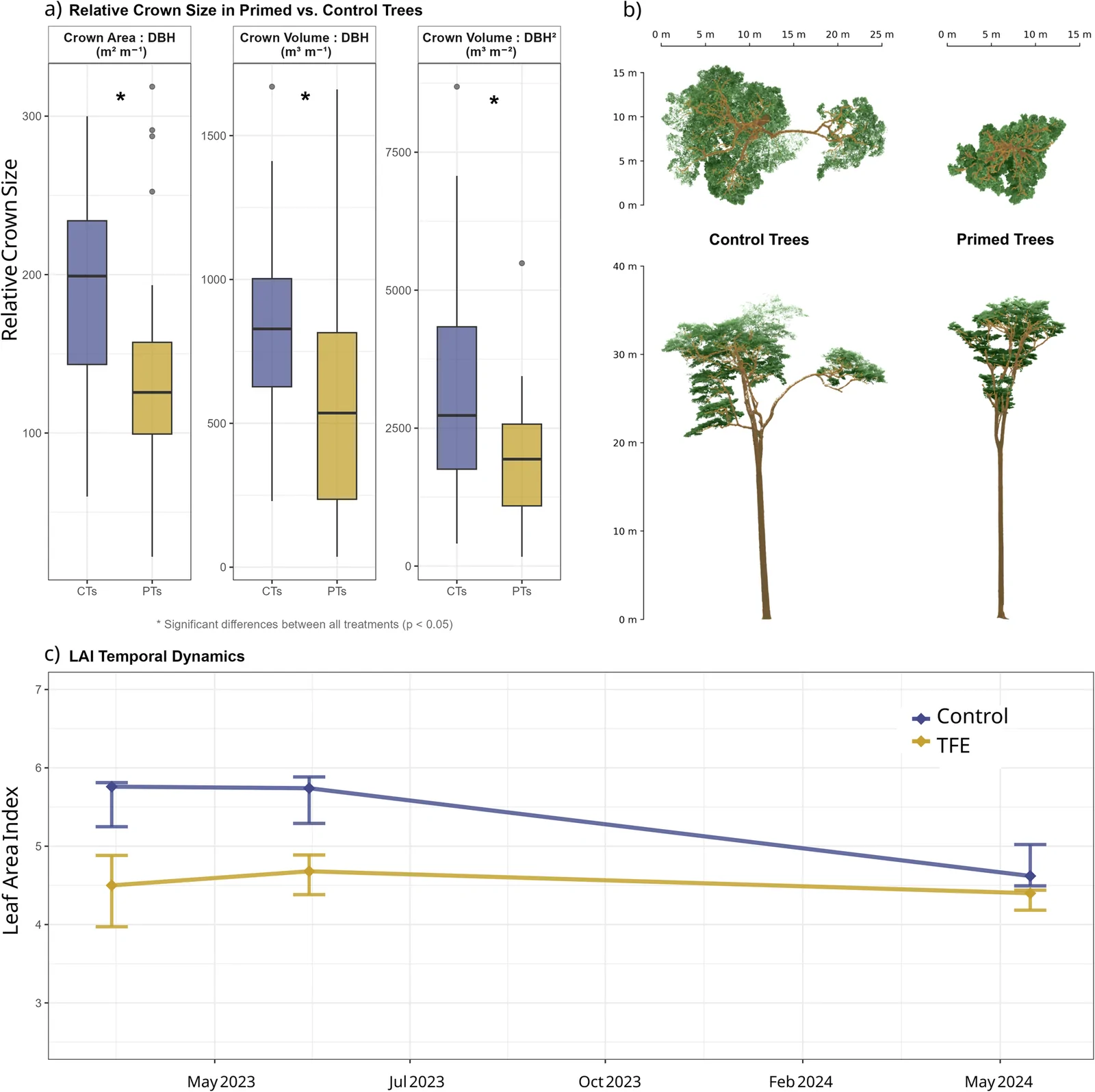
Drought-primed trees display smaller relative crowns. Structural comparison between drought-primed and control tropical forest trees. **a** Architectural metrics: relative crown size to stem size metrics calculated from terrestrial laser scanning (TLS) of the studied primed trees (PTs; goldenrod) compared to the respective control trees (CTs; deep blue) (*n =* 45): (i) Crown area normalised by stem diameter (left; one-sided *t*-test: *t =* 2.2, *p =* 0.016), (ii) crown volume per unit stem diameter (centre; one-sided *t*-test: *t =* 2.1, *p =* 0.023), and (iii) crown volume per unit stem cross-sectional area (right; one-sided Wilcoxon test: *W =* 319, *p =* 0.034). Boxplots show median, quartiles, and range; asterisks (*) indicate significance of the *p*-value at the 95 % level (*p <* 0.05). **b** Visualisation of the structural differences for two individuals from the genus *Manilkara* with similar tree height (37 m) but lower relative crown area in primed (right) vs control tree (left) derived from TLS. **c** Temporal dynamics of Leaf Area Index (LAI) from 25 measuring points per plot measured by hemispheric photography pre- El Niño drought (*n =* 25, 21), during – (*n =* 25, 11), and post- drought (*n =* 21, 24) from the Control and TFE plot, respectively. Connected points represent median values with error bars showing 95% confidence intervals. Statistical differences were tested using non-parametric Wilcoxon rank-sum tests and Kruskal-Wallis tests. TFE = Throughfall-Exclusion treatment plot.

We tested whether tree structural differences were present between PTs and CTs to further explain the observed divergence in their ability to resist and recover from the El Niño drought. Using terrestrial laser scanning (TLS) to quantify divergent canopy responses of trees between drought-exposed and control forest plots, we assessed canopy structure using pre- El Niño drought tree-level canopy structural data (*n* = 45) and complemented this analysis with temporal plot-level leaf area index (LAI, *n* = 25 per plot) measurements across three time points (pre-drought, drought (2023), and post-drought (2024), *n* = 127 measurements) to quantify divergent canopy responses of trees between drought-exposed (TFE) and control forest plots. Tree-level canopy structural data obtained from TLS supported the hypothesis that structural differences are present with regard to individual crown size, resulting in smaller transpiring surfaces in PTs compared to CTs (Fig. 3). Three complementary crown metrics demonstrated significant structural differences between treatments. (i) The ratio of individual projected tree crown area [m²] to stem diameter [m] was significantly lower in PTs with a mean of 141.0 m² m⁻¹ [95% CI = 99.4, 157.0] compared to CTs with a mean of 192.0 m² m⁻¹ [95% CI = 143.0, 234.0], as shown by a two-sample *t*-test (*t* = 2.2, df = 37, *p* = 0.016, 95% CI for difference = 4.3, 97.2). Consistent with this: (ii), crown volume per unit stem diameter was significantly smaller in PTs (mean = 585.0 m³ m⁻¹, 95% CI = 236.0, 815.0) than in CTs (mean = 844.0 m³ m⁻¹, 95% CI = 627.0, 1003.0; *t*-test: *t* = 2.1, df = 35, *p* = 0.023, 95% CI for difference = 3.8, 513.8); and (iii) crown volume relative to stem cross-sectional area, showed PTs to have significantly smaller values, approximately 70% of CTs (median values of 1938.0 m³ m⁻² [IQR = 1091.0, 2573.0] in PTs compared to CTs with 2730.0 m³ m⁻² [IQR = 1756.0, 4336.0], Wilcoxon test: *W* = 319, *p* = 0.034, Fig. 3a, b). Collectively, these results demonstrate that the overall canopy structure supporting the transpiring leaf surface area and volume was significantly reduced in PTs across multiple structural metrics. The crown area to diameter ratio suggests PTs maintain approximately 26.5% smaller crown area per unit stem diameter than CTs, indicating substantial structural adjustment in response to chronic experimental drought exposure.

In addition to the tree-level structural differences, our analysis also revealed fundamentally different temporal LAI dynamics between treatments during the extreme 2023 El Niño. The TFE plot maintained remarkable stability in LAI across the observation period (Kruskal-Wallis *χ*² = 1.55, *p* = 0.459), with median values remaining consistently between 4.40–4.59 m² m⁻². In contrast, the control plot experienced significant temporal LAI decline of about 20% in response to the acute El Niño drought (Kruskal-Wallis *χ*² = 17.25, *p* < 0.001), dropping from 5.76 m² m⁻² in July 2023 to 4.62 m² m⁻² by September 2024 (Wilcoxon signed-rank test: *p* < 0.001). Treatment differences were highly significant across all time points (Wilcoxon test: *p* < 0.001), but these differences became less pronounced over time (Fig. 3c). While the control forest initially maintained higher LAI values than the TFE pre-El Niño (July 2023: median difference = 1.26 m² m⁻², *p* < 0.001), this gap narrowed after the El Niño, with the September 2024 data showing reduced differences between treatments (median difference = 0.22 m² m⁻², *p* = 0.020). No tree mortality occurred during the measuring period, suggesting that temporal changes in gap fraction and thus LAI in the control were principally driven by leaf shedding.

**Fig. 4:**
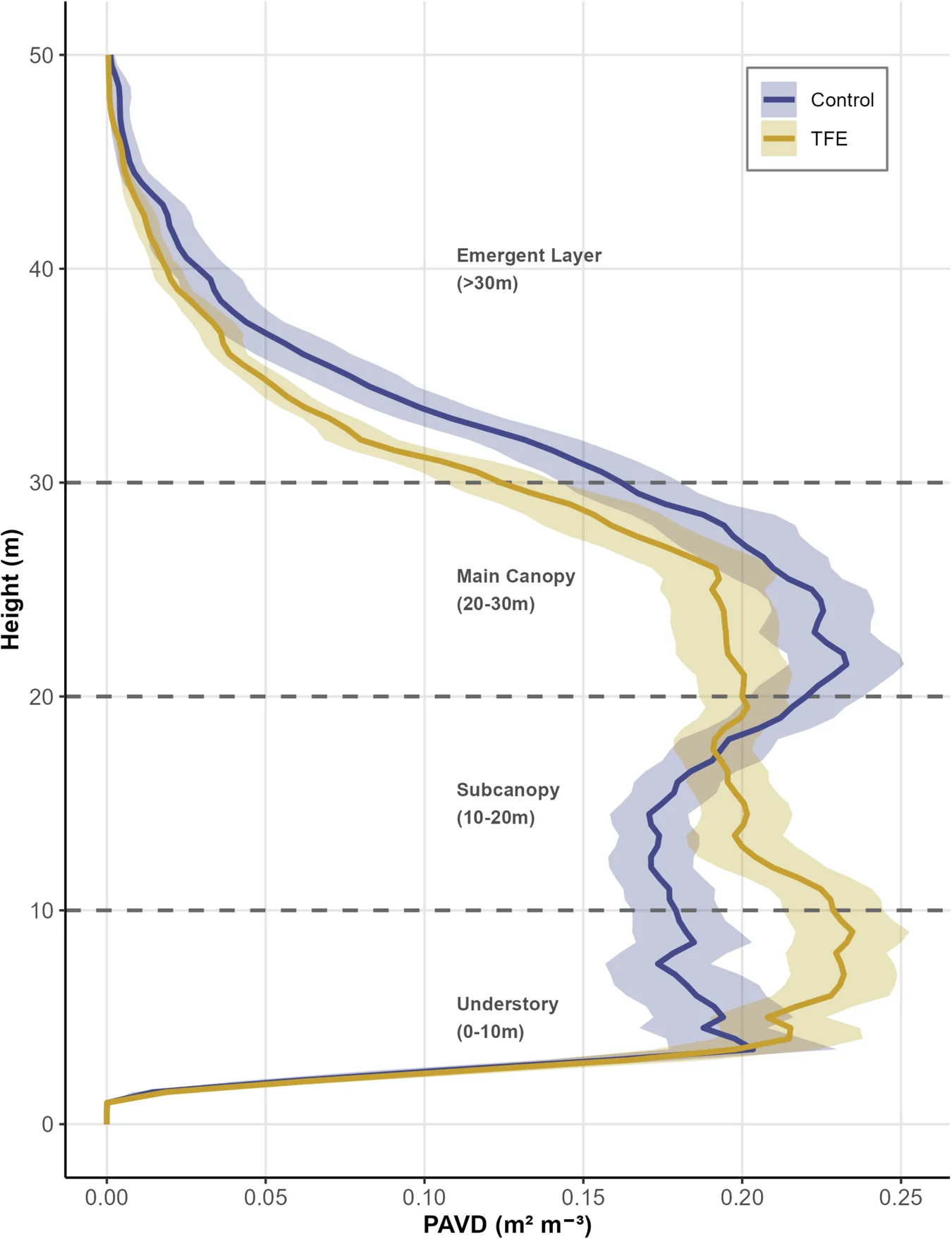
Structural differences between plots across forest strata. Plant Area Volume Density [m^2^ m^−3^] (PAVD) analysis across forest strata in long-term throughfall-exclusion experiment (TFE; goldenrod) versus control plot (deep blue), collected in October 2023. Vertical distribution of plant material density measured using terrestrial laser scanning (TLS) across 50 m height profiles. Data represent mean PAVD values (m² m⁻³) with 95% confidence intervals calculated from 36 measurement locations per plot. Forest structure was stratified into four vertical zones: Understorey (0–10 m), Subcanopy (10–20 m), Main Canopy (20–30 m), and Emergent Layer (>30 m), indicated by horizontal dashed lines and labelled regions. Statistical comparisons between treatments were conducted using two-sided Wilcoxon rank-sum tests for each stratum separately. The vertical profile illustrates the distribution of photosynthetic and structural plant material from forest floor to canopy top, enabling quantification of biomass redistribution patterns between drought-exposed and control forest conditions.

Furthermore, plot-level structural plant area volume density (PAVD, [m^2^ m^−3^]) analysis revealed significant differences in the structural organisation across forest strata between the treatment and control. Both the Understorey (ground-10 m) and Subcanopy (10–20 m height) exhibited higher density in the TFE forest canopy (Wilcoxon test: *p* < 0.08), while the upper strata of the Main Canopy (20–30 m height, Wilcoxon test: *p* < 0.001) and the Emergent Layer (>30 m height, Wilcoxon test: *p* = 0.02) showed the opposite pattern (Fig. 4). The Main Canopy showed a 14.3% lower density in the TFE forest. These findings, on individual tree and whole-forest scale, indicate a vertical re-distribution of functional plant material, with the drought-exposed forest displaying a less dense upper canopy, where atmospheric demand is likely to be at a maximum. Together, the TLS data reveal significant structural adjustments on both ecosystem and individual tree scales as responses to long-term drought exposure in the TFE, whereas the LAI data reveal structural responses to acute drought in the control.

## Discussion

**Fig. 5:**
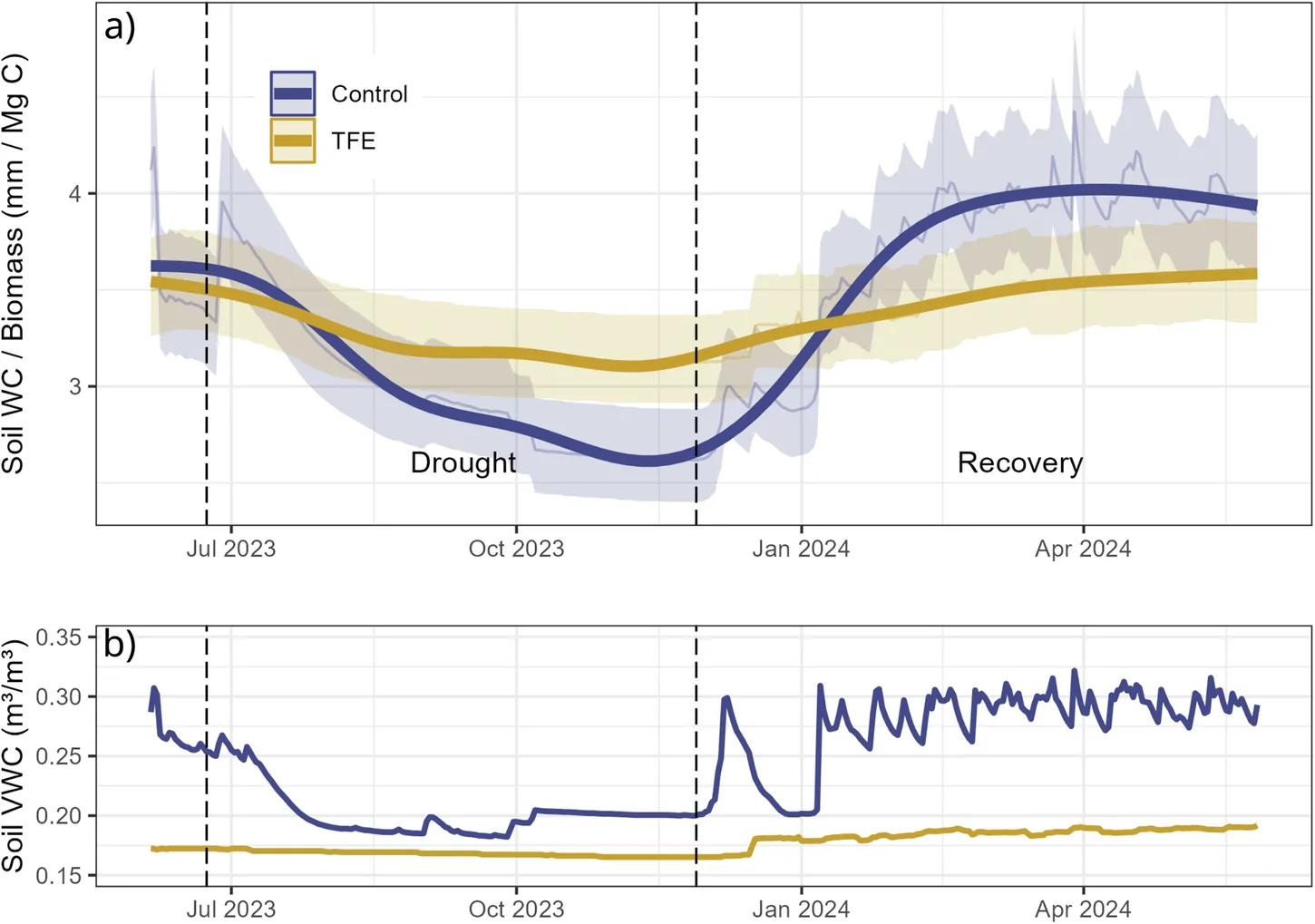
Soil moisture availability during El Niño drought. **a** Time-series of plot-level soil water content relative to the above-ground biomass [MgC/mm] in the Control (deep blue) and TFE (goldenrod) plots measured up to 4 m depth before, during and after the 2023 El Niño drought. General additive models (GAM) were used to derive the solid tendency lines, and error bands represent the 95% confidence interval of soil water content relative to biomass centred around the daily mean of each plot. Vertical dashed lines delineate drought onset (23 June 2023) and cessation (28 November 2023). **b** Time series of absolute soil volumetric water content [m^3^ m^−*3*^] per plot measured at 0.5 m depth, without accounting for the aboveground biomass and stand density.

We demonstrate that trees pre-exposed to multi-decadal recurrent soil drought (drought-primed trees, PTs) exhibited strong physiological resistance and resilience during the acute and extreme 2023 El Niño. In contrast, control trees (CT) showed considerable drought sensitivity and legacy effects in response to the acute drought (Fig. 1). The PTs fully recovered water transport rates within six months, whereas the CTs failed to recover in the post-drought rainy season despite soil water recovering to pre-drought conditions. The mechanisms underlying the observed physiological drought resistance in PTs are complex, reflecting the integration of ecosystem-scale processes, such as drought-induced tree mortality that alters soil water competition^41,44^, with tree-level adjustments including reduced crown size and leaf area, which lower atmospheric water loss and soil water demand. While treatment differences in drought resistance trajectories were primarily driven by higher soil water availability per unit biomass in the TFE plot, crown architecture further modulated resistance dynamics, indicating joint control by ecosystem- and tree-scale structure. Although absolute soil moisture was lower in the TFE, reduced biomass maintained comparable biomass-normalised soil water availability pre-drought^41^. However, during the acute 2023 drought, the control experienced lower levels of soil water availability relative to biomass (Fig. 5). This triggered stronger reductions in transpiration in CTs, together with stricter stomatal regulation and leaf shedding responses, with persistent hysteretic legacy effects after soil moisture recovery^24,45,46^. In contrast, the drought-primed system, shaped by more than two decades of intensified dry seasons, balanced water-use and demand through long-term structural adjustments without apparent lasting physiological legacy effects.

In this context, recent work identified a critical soil moisture threshold of 0.32 m^3^ m^−3^ regulating transpiration in a central Amazonian forest^47^. During the 2023 drought in Caxiuanã, control plot soil moisture remained below this threshold throughout the dry season (Fig. 5b), consistent with strong transpiration declines in CTs (Fig. 1). At peak drought, soil moisture dropped even below 0.19 m^3^ m^−3^, a threshold recently associated with rapid depletion of stem water reserves in Amazonian trees and palms as limiting soil water uptake increases reliance on internal water storage, highlighting the importance of stem water pools in buffering drought stress^48^. However, such critical soil moisture thresholds controlling transpiration can vary substantially across ecosystems, ranging from over 0.26 m^3^ m^−3^ in humid systems to 0.12 m^3^ m^−3^ in arid systems, largely due to differences in aridity, leaf area, and soil texture^49^. This contrast between systems further highlights the value of using generalisable comparators such as relative rather than location-specific soil moisture threshold analyse^41,50^.

Our results further identify structural reductions of the crown size as a significant mechanism enhancing responses to acute drought. A smaller crown relative to stem size reduces tree-level sensitivity to high evaporative demand, allowing stomata to be kept open with lower absolute water loss, with potential cooling effects on leaf and canopy temperatures^33,37,51^. In contrast, CTs with larger crowns require stronger stomatal regulation to maintain similar water potentials under greater atmospheric exposure. This is consistent with the higher water-status stringency observed in CTs despite similar minimum leaf water potentials between treatments. The absence of significant differences in minimum leaf water potential between treatments aligns with previous experimental and modelling work, suggesting that hydraulic safety can be maintained either through stomatal closure and leaf area reduction during acute drought (CTs) or through longer-term structural canopy adjustments (PTs)^33,35,36^. However, we caveat that leaf water potential was measured only bimonthly because of logistical constraints at this remote site. As the 2023 drought extended unexpectedly into late November, we were unable to sample at peak drought. Notably, work from a temperate forest TFE, using higher-frequency sampling, revealed brief but significant differences in water potential between treatments with a rapid recovery, suggesting that our lower sampling frequency may have missed this critical window of variation^24,43,52^.

Furthermore, water-status stringency depends on both VPD and leaf water potential differences^39^. While leaf water potentials were slightly, but not significantly, higher in CTs than PTs, our VPD measurements (16 and 42 m height) likely did not capture crown-level atmospheric variability associated with canopy openness and structural heterogeneity. Consequently, VPD, and thus stringency, may be underestimated for some trees in the more open TFE canopy. We acknowledge this as a limitation and note that future studies would benefit from higher-resolution vertical VPD profiling. Nevertheless, stringency incorporates the ratio of leaf conductance to hydraulic supply capacity under reference well-watered conditions $(\frac{\hat{{g}_{{tot}}}}{{K}_{L}})$, which was greater in CTs than PTs (Supplementary Fig. 4), consistent with their larger crowns and greater atmospheric exposure^33,37^. This highlights the value of null-referenced stringency metrics over traditional iso-/anisohydric classifications, which have been criticised for conflating plant hydraulic behaviour with variation in environmental conditions^39,53^. The CTs also exhibited a clear drought legacy, evident in limited transpiration recovery and substantial LAI loss. Leaf area reductions, observed during previous El Niño droughts and the initial TFE dry-down in 2002 ( ~ 20% LAI decline), provide short-term hydraulic protection^26,36,38,54,55^. However, under long-term drought, larger structural changes in the canopy appear to be favoured. Accordingly, the TFE forest had already shifted to a lower but stable LAI, whereas CTs underwent substantial leaf area loss during the 2023 El Niño to maintain hydraulic safety^38,56–58^.

Collectively, our results show that structural adjustment is a critical, yet often underappreciated, determinant of drought resistance and resilience in Amazonian trees^28,59^. Previous work has suggested that adjustments in classically assigned functional traits appear to be limited in their maximum capacity to maintain tree function and reduce mortality in individual trees and at ecosystem scale^31,32,60^, despite the predictive power of variation in these traits over larger scales across regions^30,61–63^. Many organ-scale traits, including leaf osmotic properties and xylem vulnerability, are evolutionarily conserved and therefore unlikely to shift rapidly in response to drought^61,64^. The response to strong variation in long-term drought stress is likely to be explained far better by the inclusion of structural adjustment, which has been noted elsewhere to be a key process that governs drought responses^35,46,52^. Because terrestrial laser scanning was not available at the start of the experiment, uncertainties remain regarding the pre-drought structural state and temporal development of the TFE forest. Consequently, it is unclear whether the current phenotype of PTs reflects structural plasticity or selective mortality. Nevertheless, as structural shifts become more prevalent with climate change, they carry important implications for above-ground biomass estimates, which typically do not account for canopy degradation. Allometric models may increasingly underestimate biomass losses in tropical forests as climate change progresses, potentially masking early signs of structural decline and ecosystem change^65,66^. This highlights the value of laser scanning technologies for improving biomass estimation^67^. Our findings also carry implications for remote-sensing observations of these forests, as canopy dieback affects canopy roughness, biomass and moisture, which have been linked to declines in radar backscatter and may become an increasingly common signal in drought-affected forests^56–58^. Future work should determine whether the TFE forest now resembles naturally drier Amazonian forests and investigate the mechanisms driving crown reduction, including hydraulic branch failure, cladoptosis, and constrained regrowth under chronic drought^54,61^. Future work should also address (epi)-genetic modifications and the potential for transgenerational memory effects to better inform our understanding of the decades-to-century-scale resilience of forests to climate stress^42,68^.

Our study highlights the importance of linking short- with long-term drought responses^27,52^. Ecosystems are not only impacted by current climate, but their growth and environmental conditions are shaped by antecedent climate. Such lagged responses are known as memory effects and can influence the impact of climate extremes on ecosystem functions^69–71^. In this context, we argue that the overall allometry of the PTs and the structure of the TFE forest carries a multi-decadal-scale effect of the drought treatment. That is, the drought-primed system harbours an ‘ecological memory’ of recurrent intensified dry season water limitation, and this is imposed at the intersection of physiology and structure, so that PT function is affected favourably under future extreme drought, leading to the observed increased resistance and resilience even in extreme conditions such as the recent 2023 El Niño^42,68,72^. Our findings may seem to challenge previous assumptions that prolonged drought legacies could ultimately manifest as retained and accumulated hydraulic damage leading to reductions in forest resilience according to alternative stable state theory (ASST)^5,73^. Remote sensing evidence of changes in biomass using vertical optical depth (VOD), including critical slowing-down analyses, has been interpreted as early warning signals of critical transitions and loss of resilience in Amazonia^4,5,56,58^. However, many resilience proxies (e.g., critical slowing down) are coarse and may not capture such fine-scale ecological mechanisms driving recovery^4,58^. Although biomass reductions from mortality and canopy degradation are detrimental and reduce productivity and carbon storage, they may also alter competition for water and other resources in ways that enhance the survival and relative performance of remaining trees^41^. Thus, our results complement recent theoretical frameworks by highlighting that resilience trajectories may be context dependent and strongly mediated by system-specific biological mechanisms that are often underrepresented in top-down climate resilience approaches, including acclimation, structural adjustment, mortality-driven reorganisation, and shifts in competitive interactions which may alter hydraulic vulnerability^74^. While hydraulic legacy effects and hysteresis remain important components of acute drought responses^45^ (as we show in the control), our findings suggest that long-term exposure can simultaneously promote compensatory adjustments with large effects that buffer surviving trees against future extremes.

Climate change will continue to disrupt biosphere–atmosphere interactions in the Amazon, and forest resilience will depend on the capacity for adjustment to sustained ecological stress beyond individual events^46,52,69^. These longer-term adjustments can strongly reshape projected resilience trajectories but are rarely included in dynamic vegetation models used to assess future forest–climate feedbacks^70,75^. We provide evidence here that the emergent response to long-term drought in Amazon rainforest trees includes an imprint operating over timescales that alter structure as well as physiological processes^46,52,71,72^. This changes our understanding of the decadal-scale response by Amazonian forests to climate change, suggesting that a previously unreported resilience to extreme events occurs in trees in response to long-term drought, with ecosystem-scale effects. However, these adjustments entail substantial costs, including reduced productivity, lower above-ground biomass, and diminished ecosystem services, but ensure survival and increase the competitive advantage of surviving trees under a rapidly changing and drier climate^41,65,66^. These outcomes contribute to the discussion on tipping points and state-transition regarding the response by Amazon forests to climate stress, in support of the possibility of intermediate states and highlighting a dynamic resilience trajectory in these ecosystems^5,6,41,74^.

## Methods

### Site

The study was conducted in an Amazonian lowland tropical rainforest situated in the Caxiuanã National Forest, Floresta Nacional de Caxiuanã, state of Pará, in northern Brazil (1°43′S, 51°27′W). The site is designated as an old-growth *terra firme* forest and is located within the Xingu–Tocantins–Araguaia moist forests ecoregion. The seasonally dry evergreen forest receives an annual precipitation of between 2,000 and 2,500 mm, with monthly rainfall dropping below <100 mm month^−1^ during the dry season (August—November, Supplementary Fig. 2). The equatorial climate results in steady temperatures throughout the year with a mean below-canopy temperature of 26 °C. The experiment consists of two 1 ha plots.

### Throughfall exclusion

A one-hectare plot constitutes the through-fall exclusion treatment (TFE) in this experiment, where 50% of the incoming rainfall is deflected from the soil via a system of plastic panels and gutters installed 1–2 m above the ground. The plot was trenched with a depth of 50–100 cm around its borders to avoid water entering the plot laterally through root or microbial connections. The large size was necessary to implement a manipulation study at a scale that considered a whole stand of trees, rather than a few individuals^26,27,40,41,76,77^. With a distance of 50 m from the TFE and on similar soil and slope, a respective control plot was also installed, without TFE panels but with respective trenching. While the TFE treatment was maintained continuously throughout each year, the net effect of the TFE, a partial throughfall exclusion, was to simulate the recurrence of an El Niño-type soil drought by exacerbating dry season water limitation. This occurred for >20 consecutive years by increasing the length and intensity of each dry season. During the wet season, however, rainfall remained sufficient—monthly totals exceeded 100 mm even with 50% exclusion—allowing for partial recovery (Supplementary Fig. 2). Thus, trees in the TFE plot experienced pronounced seasonal drought stress rather than year-round water limitation. This is supported by data from this study site that show no differences in wet-season transpiration flux between plots, whereas a pronounced reduction of dry-season transpiration in the TFE compared to the control^77^, and wet-season growth and respiration data suggesting wet-season hydraulic recovery^78^. Collectively, this suggests that trees in the TFE were indeed primed to annual cycles of soil-water limitation during the dry season, rather than suffering persistent water stress^42^. The experiment has been maintained since its installation in January 2002.

### Sampling design

We sampled 20 Amazonian trees in the TFE and 20 in the control (*n* = 40) from May 2023 until July 2024, capturing the historic drought during the El Niño 2023. Sampling was designed to minimise taxonomic and size-related bias by selecting individuals from comparable species or, where not possible, congeneric taxa and similar size classes across plots (Supplementary Table 5). However, due to differences in species composition and availability between plots, exact taxonomic matching was not always achievable. The resulting dataset therefore represents a partially stratified, taxonomically constrained sampling design rather than a fully paired design. Our models incorporate taxonomic identity as a grouping term. Previous work at this field site found little evidence for functional divergence between the control and experimental plots, suggesting that long-term drought exposure did not substantially alter community-level functional composition^31,32^. Consistent with this, we measured functional traits related to plant economic strategy, drought tolerance, and tree size — including wood density, leaf water potential at turgor loss point, and DBH—and did not find significant differences between plots among the sampled individuals, suggesting that interspecific functional variation likely had only a minor influence on the observed resistance and resilience dynamics.

### Meteorological data

The plot was equipped with a meteorological station installed on a ~45 m tall tower. We measured data on precipitation, air temperature (*T* [°C]) and relative humidity (RH in %). Sensors for T and RH were installed above (42 m) and below the main canopy (16 m). Vapour pressure deficit (VPD) was calculated from T and RH using the bigleaf package^79^. Additionally, we calculated the Standardized Precipitation Index (SPI) using the SPEI package^80^. We integrated the drought index over a 3-month period, SPI-3, which has been recommended for characterising meteorological drought and soil water deficits in tropical ecosystems^81^. We used the long-term monitoring data on precipitation at the site, dating back to 1982, to calculate the mean precipitation for the dry season between August and November. We then calculated the departure of each year’s dry season from the long-term mean, excluding the extreme year of 2023 (Supplementary Fig. 1). To assess the severity of 2023 drought conditions, we computed seasonal means for daily maximum temperature, daily maximum vapour pressure deficit (VPD), and daily minimum relative humidity for each year in the historical baseline period (2001–2022) and compared these with the 2023 seasonal mean. For each climate variable, we calculated the empirical percentile rank of the 2023 seasonal mean within the 22-year baseline distribution. Percentile rankings indicate where 2023 conditions rank relative to the historical record, providing interpretable effect sizes for the observed climate anomalies.

### Soil water content per unit biomass

We calculated soil water content (SWC) for the upper 4 m of the soil profile using permanently installed soil access pits located in both control and throughfall-exclusion (TFE) plots. Volumetric soil moisture was recorded at hourly resolution using time-domain reflectometry (TDR, CS616, Campbell Scientific) installed at depths of 0, 0.5, 1, 2.5 and 4 m. These depths were selected to capture the majority of the fine-root distribution and therefore the bulk of plant-accessible water in the system. To derive a profile-integrated estimate of water storage, the soil column was partitioned into depth intervals (0–0.5, 0.5–1, 1–2.5, and 2.5–4 m), and volumetric water content in each layer was converted into absolute water storage by multiplying by the corresponding soil volume per hectare (10,000 m² × layer thickness). Layer-specific water storage values were then summed to obtain total soil water content for the upper 4 m of the profile (expressed as L m⁻² or mm). We calculated the water content per unit biomass by dividing the maximum absolute water content in the first 4 m of depth by the annual biomass. To estimate aboveground biomass (AGB), we used mean wood density (WD) and higher and lower 95% confidence intervals, tree height (H) and stem diameter at breast height (DBH) using allometric models, following previous implementations in the same site.

The following allometric equation was used to calculate tree height from DBH: $H=227.35\left(1-\exp \left(-\,0.139\,{{DBH}}^{0.5550}\right)\right).$

Then, we applied a recently derived allometric equation from TLS data in 2018, using the measured DBH (m), the previously estimated H (m) and WD (g cm^−3^) taken from the literature. This allometric equation took the form:

1 $${AGB}=0.088({{{WDH}\left({DBH}\right)}^{2}})^{0.954}$$

For more details, refer to the methods of Sanchez-Martinez (2025)^41^.

### Sap flux

Sensors (EMS81; Environmental Monitoring Systems, Brno, Czech Republic, http://www.emsbrno.cz) were installed on the south side of the focal trees, and programmed to log data in 15 min intervals for high temporal resolution. The selection of trees was stratified by size to ensure a comparable diameter distribution to that observed in the plot^82^. The EMS81 sensors use the heat balance method^77,83,84^. Data on total tree sap flux (kg h^−1^) were collected between May 2023 and July 2024 and aggregated on a daily basis (kg day^−1^). Values of sap flow consistently showed an offset from zero due to a constant portion of heat loss from the heated electrodes being conducted into the xylem tissue. To baseline the data, 10% quantile regressions were performed for each individual setting hourly sap flow as a response variable and time as a predictor and setting the ‘tau’ argument to 0.1^41^. Models for each individual were then used to predict values for the period of study. Finally, predicted values were subtracted from the raw data. Daily variations of sap flow in the wet season before the El Niño were large, likely due to cloud cover and rainfall, and to reduce this variation, the rolling daily mean flux was calculated within a five-day interval.

### Leaf data

Water potential (*Ψ*) was directly measured in the field immediately after collection using a pressure chamber (Model 1505D, PMS; 0.05 MPa resolution). Predawn (*Ψ*_pd_) measurements were taken between 3:30 – 5:30 A.M, and Midday (*Ψ*_md_) measurements were conducted on the same day between 11:30 A.M. – 1:30 P.M. Trees were climbed and outreaching branches were cut from the upper third part of the tree crown. For each tree, we measured *Ψ* of two leaves, or three leaves if the first two measures differed by more than 0.2 MPa for *Ψ*_pd_ and 0.4 MPa for *Ψ*_md_ measurements. Measurements were conducted bi-monthly during five campaigns from May 2023–May 2024. Finally, the mean of the two repetitive samples was taken. Data from the campaign in December 2023 were excluded from the analysis, as the largest trees could not be sampled due to unfavourable weather conditions.

### Plant water status stringency

We used a stringency metric to quantify plant water status regulation (*S*^WS^; for a more detailed description see Mencuccini et al. (2024))^39^. For these calculations, time-series data of VPD (measured at two different canopy positions; above canopy: 42 m and below canopy 16 m), *Ψ*_pd_ and *Ψ*_md_ were necessary, where *Ψ*_pd_ acts as a surrogate for Ψ_soil_. We acknowledge that *Ψ*_pd_ might not be equivalent to *Ψ*_soil_ and may deviate under conditions of incomplete nocturnal equilibration or residual night-time transpiration; however, it was used here as a practical proxy for plant-accessible water status given the absence of spatially resolved soil water potential measurements across rooting depths. For more details, refer to Mencuccini et al. (2024). Tree-level height estimates from terrestrial laser scanning (see below) were used to match trees to their respective VPD exposure, where trees below the mean tree height (26.5 m) of the subcanopy VPD measurement were matched with VPD at 16 m. Trees with a height >27 m were matched with above-canopy VPD measurements. The 27 m height threshold is further justified by PAVD data (Fig. 4) from the Main Canopy layer (20–30 m) showing a clear structural transition from high crown density to sparse emergent layer between 26 and 27 m in both plots, where PAVD above 27 m falls outwith the 90^th^ quantile of the maximum PAVD, suggesting that canopy maximum is at 27 m. The metric for total S^WS^ allows for discrimination between regulation against the soil (S_Ψ-soil_) and against atmospheric forcing (S_VPD_, Supplementary Fig. 3). The stringency metric works under the assumption of steady-state water balance.

2 $${\varPsi }_{{leaf}}={\varPsi }_{{soil}}-\frac{{g}_{{tot}}}{{k}_{L}}{VPD}$$

where$\,\frac{{g}_{{tot}}}{{k}_{L}}$ describes the variability of total leaf vapour conductance (sum of stomatal andboundary layer conductance) and the whole-plant hydraulic conductance divided by leaf area, and is defined as:

3 $$\frac{{g}_{{tot},i}}{{K}_{L},i}=\frac{{\varPsi }_{{soil},i}-\,{\varPsi }_{{leaf},i}\,}{{VPD},i}$$

The upper distribution of these data from our time series then represents the reference point, under non-stressed, well-watered conditions,$\,\frac{\hat{{g}_{{tot}}}}{{K}_{L}}={quantile},\,95({pdf}\left(\frac{{g}_{{tot},i}}{{K}_{L},i}\right))$ (Supplementary Fig. 4). Note that the 99^th^ quantile of that distribution is favoured as a reference point when sampling number is high. However, due to low temporal sampling number for each individual tree during our study, we aggregated the hydraulic conductance by plot treatment. However, to avoid overestimation of stringency, driven by larger values of $\frac{{g}_{{tot},i}}{{K}_{L},i}$ of few trees, we used the 95th quantile as more conservative upper distribution rather than the 99^th^ quantile (Supplementary Fig. 4). To ensure that stringency differences were not overestimated, we calculated stringency using the 99^th^, 95^th^ and 90^th^ quantile. However, even with the most conservative approach (90^th^ quantile), the significant differences in stringency between plots during the drought remained.

The hydraulic effects of the external environmental forcing, that is from VPD from the atmosphere and ${\varPsi }_{{soil}}$, are then defined as:

4 $${\varPsi }_{{hf},i}={\varPsi }_{{soil},i}-\frac{\widehat{{g}_{{tot}}}}{{K}_{L}}{VPD}$$

The departure of the measured ${\varPsi }_{{leaf}}$ from the environmental effects ${\varPsi }_{{hf}}$ then define the stringency of water status regulation by the plant:

5 $$\,{{{\boldsymbol{S}}}}_{{{\boldsymbol{i}}}}^{{{\boldsymbol{ws}}}}={{{\boldsymbol{\Psi }}}}_{{{\boldsymbol{leaf}}},{{\boldsymbol{i}}}}-{{{\boldsymbol{\Psi }}}}_{{{\boldsymbol{hf}}},{{\boldsymbol{i}}}}$$

### Drought resistance and resilience

We calculated physiological resistance (*Rt*) and resilience (*Rs*) for tree-level transpiration [kg day^−1^] according to Lloret, et al. (2011)^23^. We quantified the *Rt* for each individual tree, that is, its ability to limit the reduction in transpiration during drought, as the ratio between its transpiration during- (Dr) and pre-drought (PreDr). The PreDr period was defined as the period in the peak wet season from the 01st of May to the 15th of June 2023 (Supplementary Figs. 1, 2).

6 $${Rt}=\frac{{Dr}}{{mean}({PreDr})}$$

We further quantified *Rs*, that is the capacity of trees to reach pre-drought transpiration levels, estimated as the ratio between performance post- (PostDr) and pre drought (PreDr)

7 $${Rs}=\frac{{PostDr}}{{mean}({PreDr})}$$

PreDr was calculated as the mean of daily transpiration over a one-month period. Full recovery was assumed when the resilience value stayed above 0.95 PreDr for 5 consecutive days post-drought.

### Drought Period

The El Niño caused an abrupt change from wet to dry season. The drought onset was defined as the day from which precipitation dropped to continuously below 10 mm day^−1^ (with two days of exception), and the drought breaking was defined as the day where rainfall raised to >20 mm of daily rain (23rd of June–28th of November, Supplementary Fig. 5). Thus, the TFE panels did not notably affect the drought onset. We could neither detect an effect of the panels on the drought onset between plots in the soil water content data (Fig. 5) nor stem water content data (see Supplementary Fig. 6), measured using FDR sensors calibrated for woody tissue (see Martius et al. (2024)^85^). These data show that trees from both plots were equally sensitive to the above-defined drought onset and return of rainfall date, with no detection of lag effects between plots.

### Leaf area index

Hemispherical Photos were taken from 25 equally distanced positions within a grid of each 1 ha plot at dawn from 6 AM at a height of 1.5 metres from the forest floor during three campaigns: pre-drought, during drought (July & October 2023) and post-drought (September 2024) (total *n* = 127). The hemispheric photos were pre-processed and analysed using the HemispheR package in R^86^. Gap fraction was analysed using the gap.frac2 function, with the number of zenith rings set to five. This setting is suggested for comparability with LAI-2000/2200 Plant Canopy Analyzer, which was used for LAI estimation earlier in this experiment^26^. With this setting, the effective leaf area (*Le*) is comparable with the apparent LAI derived from LAI-2000/2200. *Le* was calculated from the logarithm of the arithmetic mean gap fraction. Out of the total of 150 photographs taken, 23 were removed after a quality check of the quality of pictures taken, where overgrowing leaves or poor light conditions resulted in strong outliers in the dataset that made their inclusion unreasonable. For consistency with the literature, including from this experiment, we use the leaf area index (LAI) as a function of height to formulate the equations. However, it is important to note that this is essentially the plant area index (PAI), which is interpreted as the one-sided area of plant material surface per unit ground surface area.

### Terrestrial Laser Scanning

TLS (terrestrial laser scanning) data were collected with a RIEGL VZ-400i terrestrial laser scanner in October 2023. The VZ-400i has a laser wavelength of 1550 nm and 0.35 mrad beam divergence. The angular resolution used was 0.04°, and the pulse repetition rate was 600 kHz. Scanning was done on a 10 m × 10 m grid, with two scans (one vertical and one horizontal) taken at each position totalling 242 scans per plot. To minimise the effect of wind on the data, scans were not executed when wind was strong enough to move the woody parts of the trees. TLS data were co-registered, and individual trees were manually segmented in RiSCAN PRO (RIEGL Laser Measurement Systems GmbH, Horn, Austria, version). Individual tree metrics were derived from segmented point clouds using the R package ITSMe v1.0.0, which included tree height, DBH, crown area (concavity) and volume (alpha)^87^. In order to estimate the crown metrics, we split up the trunk from the crown. To detect differences in relative crown size in trees between plots, we calculated the ratio of the crown area to DBH, crown volume to DBH and crown volume to stem cross-sectional area where *A* = π * (DBH/2)² for each of the trees from this study. Vertical plant area index (PAI) profiles were derived from TLS scans performed at 121 locations. At each scan location, two scans were collected: one upright and one tilted 90° from vertical, to capture the full hemisphere. Plant profiles were computed for each scan location using the solid-angle-weighted normalised method described in Calders et al. (2014)^88^, implemented in the pylidar Python package (https://github.com/armstonj/pylidar-tls-canopy).

### Statistical analysis

All data processing and statistical analyses were conducted in R 4.4.1^89^. The datasets were temporally divided into the pre-drought (<22/06/2023), drought (23/06–27/11/2023) and post-drought period (>28/11/2023) as defined above. We used linear mixed-effects models (LMMs) to analyse temporal physiological resistance (*Rt*) dynamics during the drought period, and resilience (*Rs*) dynamics during the post-drought period, with time, treatment (plot), log-transformed crown volume, and their interactions as fixed effects, and individual tree identity as a random intercept effect to account for repeated measures within trees. The model goodness-of-fit was reported as the marginal ${R}_{m}^{2}$ for the proportion of variance explained by the fixed effects only, and the conditional ${R}_{c}^{2}$ to show how much variance was additionally explained by the random effect, conditional on the fixed effects. The model included *Rt* as the response variable, while the drought-exposure time, plot treatment and crown size (l*og*(volume crown [m^3^])) were included as fixed effects. To assess potential temporal autocorrelation in the time series structure of the data, we additionally fitted linear mixed-effects models with a first-order autoregressive correlation structure (corAR1) using the nlme package. These models produced highly consistent effect estimates compared to the primary LMM framework, although statistical significance became slightly more conservative but still significant. The primary linear mixed model presented includes tree ID as a random factor, accounting for the fact that time is nested within the individual and measurements within each individual are more similar to each other than across individuals. Given that the rolling mean transformation of the response variables introduces non-independence between adjacent time points, effectively inflating short-term temporal autocorrelation, and given that the results converge with the LMM, we present the simpler LMM results as the primary analysis. Statistical summary tables of the linear models were produced and are included in the Supplementary Information. The slopes of the plot-specific linear models represent the rate of the daily resistance loss (during *Dr*)—and the rate of daily increasing resilience (*PostDr*). We computed the mean resistance at the peak of drought from estimated marginal means of the linear mixed model. While GAM models were used to display the data, simpler linear mixed models were fitted to calculate all model estimates. All coefficients were reported with their respective 95 % confidence interval. We additionally tested alternative models including soil water content per unit biomass as a covariate to assess whether treatment effects on resistance and resilience were mediated by soil moisture availability. This extended model included soil water content per unit biomass for each plot as a fixed effect alongside the core model terms.

To verify the robustness of our resilience findings, we performed bootstrap resampling analysis with 1000 iterations, randomly sampling trees with replacement from each plot and refitting the two linear mixed models (during drought and post-drought) to each bootstrap sample. This approach tested whether the observed treatment effects and temporal interactions remained consistent across different combinations of trees, addressing potential concerns about sample size limitations. The bootstrap analysis confirmed that our key findings were statistically robust, with the plot × time interaction effect remaining significant in 94.3 % of bootstrap samples for resistance, and the plot effect remaining robust in 71.5% of bootstrap samples for resilience (Supplementary Fig. 7).

Furthermore, linear mixed models were used to test for differences in plant water status stringency (S^ws^), using the same fixed terms as noted above (except crown volume) and tree ID as the random effect. One-sided t-tests were performed to test differences between the individual tree crown size metrics between CTs and PTs. The t-test assumptions were checked. When these were violated (e.g., non-normality for the metric crown volume to cross-sectional area ratio), non-parametric Wilcoxon tests were used. Temporal changes in LAI within each treatment were assessed using Kruskal-Wallis tests, with post-hoc pairwise comparisons conducted using Wilcoxon signed-rank tests to identify significant changes between time points. Treatment differences in LAI at each time point were evaluated using Wilcoxon rank-sum tests due to non-normal data distribution. Structural differences in plant area volume density (PAVD) between treatments across forest strata (Understorey (0–10 m), Subcanopy (10–20), Main Canopy (20–30 m), Emergent Layer (>30 m) were analysed using two-sided Wilcoxon rank-sum tests between plots. All data were checked for normality using the Shapiro-Wilk test. To check the assumptions for homogeneity of variance, *F*-tests were conducted. We fitted linear mixed-effect models with the ‘lmer’ function of the lme4 package^90^ and linear fixed-effect models with the ‘lm’ function (base packages). We validated model assumptions using diagnostic plots for normality (QQ-plot) and homogeneity of residuals.

## Supplementary information

Transparent Peer Review file

Supplementary Material

## Data Availability

The minimum dataset needed to replicate the analyses is available via GitHub https://github.com/lionmartius/ecomemory. This includes datasets on meteorology, leaf area index and physiological measurements of sapflow and water potentials.

## Code availability

The code used to replicate the analyses is available via GitHub https://github.com/lionmartius/ecomemory. The code was written in R- language and was organised in an R-markdown (rmd) script, which contains all code relevant to this study.
