# Supplementary material for: Amazonian trees under long-term drought exposure are more resilient to El Niño extreme
Source: Commun Earth Environ. 2026 Sep 14;7(1):738. doi: 10.1038/s43247-026-03887-0 (PMC13574715; doi:10.1038/s43247-026-03887-0)
Supplement: Supplementary file 1 [file 43247_2026_3887_MOESM1_ESM.pdf]

nature portfolio

# Peer Review File

# Amazonian trees under long-term drought exposure are more resilient to El Niño extreme

Corresponding Author: Dr Lion Martius

**This file contains all reviewer reports in order by version, followed by all author rebuttals in order by version.**

Version 0:

Reviewer comments:

Reviewer #1

(Remarks to the Author)
Review of the manuscript entitled “Amazonian trees under long-term drought exposure are more resilient to El Niño extremes” by Martius et al.

In this paper, the authors show that the eco-physiological resilience and resistance of trees pre-exposed to experimental drought for more than 20 years (PT) are higher than those of untreated controls (CT) in response to the 2023 El Niño drought.

I find this study original and highly relevant. The use of a long-term throughfall exclusion experiment provides a valuable and a rare opportunity to assess how prolonged drought exposure shapes forest resilience and resistance to drought. The analyses are solid, statistically robust, and supported by multiple lines of evidence. Overall, I have no major concerns and support the publication of this work. I do have a few minor comments and suggestions for clarification.

General comments

I wonder whether the presence of plastic panels in the throughfall exclusion setup may influence soil evaporation. Could this contribute to preserving soil moisture, or is this effect negligible? A brief discussion would be helpful.

Could vapor pressure deficit (VPD) differ between CT and TFE plots, given the differences in evapotranspiration you report? This potential discrepancy might be worth discussing as, if relevant, it can influence the plant water status stringency calculation.

Minor comments

P3, l43: “Amazônia” - portuuese ?

P3, l52: Consider noting that the influence of the NAO on Amazonian drought is relatively minor compared to ENSO.

P3, l54: Please cite more recent references. Also, Cai et al. (2014) focuses only on ENSO rather than NAO. My understanding is that the 2005 and 2010 droughts were primarily associated with warmer SSTs in the tropical North Atlantic, shifting the ITCZ northward, rather than NAO influence per se.

P12, l294: This sentence is somewhat confusing and appears to contradict your main findings:
“Both plots had similar soil-water availability on a tree biomass basis at the start of and following the drought, consistent with eco-hydrological stabilisation.”
This seems at odds with your explanation that increased resistance in PT trees arises from reduced competition for soil water following long-term structural adjustments. I’m guessing you are comparing conditions before vs. after the 2023 drought within each treatment, rather than between treatments?

P14, l349: Is species composition similar between plots? It would be nice for the reader to clarify the tree sampling strategy between CT and PT plots. In particular, could differences in species composition (e.g., a higher proportion of drought-

tolerant species in PT plots) partly explain the observed differences in resilience? It's discussed in the method section but it would be useful in the discussion (which is before the method)

P18–19 (Soil water content per unit biomass):
Please provide more detail on how soil water content was measured down to 4 m depth.
Ensure consistent use of acronyms between the text and equations (e.g., H for height), and avoid including units within equations.

P20:
Would it be more appropriate to estimate soil water potential (ψ_soil) from measured soil water content and soil characteristics, rather than relying on predawn leaf water potential (Ψ_pd)?

Reviewer #3

(Remarks to the Author)
Main Comments
I appreciate many aspects of this manuscript. It investigates the resistance and resilience of Amazonian trees under the unprecedent 2023-24 El Niño-driven drought. This is an important contribution, as most studies in the Amazon have focused primarily on drought resistance, whereas the integration of resistance and resilience is essential for ecophysiological understanding and modeling efforts. However, the presented findings raise two important conceptual issues that I encourage the authors to address more explicitly:

Paradox #1 -> Primed trees (PT) exhibited lower declines in transpiration during drought progression and higher recovery compared to control trees (CT). The authors suggest that "resilience to extreme events can emerge from long-term drought exposure through structural adjustments at both ecosystem and individual tree scale". This is a strong result and is consistent with empirical practices such as the acclimatization or "hardening" of seedlings before their transfer from nursery environments to field conditions. However, these findings appear to contrast with theoretical frameworks such as alternative stable state theory (ASST). Hammond (2020) (https://doi.org/10.3389/ffgc.2020.560409), for example, suggests that trees surviving prolonged drought may retain hydraulic damage, which can reduce resilience. In that framework, legacy effects of hydraulic failure can prevent full recovery to pre-drought physiological states and increase the probability of ecosystem transition. Similarly, Flores et al. (2024) (https://www.nature.com/articles/s41586-023-06970-0) highlight that ecosystems approaching tipping points often lose resilience even before a state shift occurs. Hysteresis dynamics are also commonly associated with such processes (e.g., Bretfeld et al., 2018 https://doi.org/10.1111/nph.15071; Brum et al., 2026 https://onlinelibrary.wiley.com/doi/full/10.1111/pce.70498 ; Gimenez et al., 2024 https://doi.org/10.1016/j.agrformet.2024.110052).
In this context, I recommend that the authors explicitly discuss how their findings relate to, complement, or potentially challenge these frameworks. In particular, it would be valuable to clarify whether the observed responses represent a form of enhanced resilience through a potential structural reorganization in terms of plant hydraulics, rather than contradicting existing theory.

Paradox #2 (Role of species composition and functional traits)

The study compares 40 trees (20 per plot) and reports higher resistance and full recovery in PT, versus lower resistance and limited recovery in CT (Figure 1). These differences are attributed primarily to structural traits such as crown size, LAI, and PAVD (Figures 3 and 4).
However, it remains unclear to what extent species composition may contribute to these patterns. The observed patterns emerge from a selected set of individuals, and interspecific variation in functional traits could partially influence the reported differences. For example, if primed trees are dominated by faster-growing, low wood-density species, they may have greater sapwood area and higher internal water storage (capacitance), which could "apparent enhance" both drought resistance and recovery. Additionally, Figure 2 suggests that control trees may reach more negative midday leaf water potentials during peak drought and higher recoveries in predawn leaf water potentials (panel C), which may indicate higher drought tolerance or a more isohydric regulation strategy (see Maréchaux et al., 2015, doi: 10.1111/1365-2435.12452).
I strongly recommend that the authors provide a table (main text or supplementary material) listing the sampled species, along with key attributes such as DBH, wood density, and any available functional traits. This would greatly improve the interpretability of the results. Furthermore, a brief discussion of stem capacitance and internal water storage would strengthen the physiological interpretation of resistance and resilience.

Specific comments

Methods Section -> Please include a table (main or supplementary) with the list of sampled species list and relevant functional traits (e.g., DBH, wood density, growth rates if available). This will help the readers to better interpret the observed patterns.

Figure 1 -> A recent study has identified soil moisture thresholds controlling transpiration across Amazon forests. In particular, sap flow responses from multiple species indicate a critical threshold in soil moisture at approximately 0.32 $m^3$ $m^{-3}$ (at ~0.5 m depth), below which transpiration declines rapidly. (see: Chambers et al., 2025,

https://doi.org/10.1038/s41586-025-09728-y). In your study, a similar threshold-like behavior appears to be present in Figure 1, where hydraulic function declines in both treatments, with a slight delay between them. I encourage the authors to explore and discuss this potential threshold response, as it may provide additional mechanistic insight into the observed dynamics.

**Open Access** This Peer Review File is licensed under a Creative Commons Attribution 4.0 International License, which permits use, sharing, adaptation, distribution and reproduction in any medium or format, as long as you give appropriate credit to the original author(s) and the source, provide a link to the Creative Commons license, and indicate if changes were made.
In cases where reviewers are anonymous, credit should be given to 'Anonymous Referee' and the source.
The images or other third party material in this Peer Review File are included in the article's Creative Commons license, unless indicated otherwise in a credit line to the material. If material is not included in the article's Creative Commons license and your intended use is not permitted by statutory regulation or exceeds the permitted use, you will need to obtain permission directly from the copyright holder.
To view a copy of this license, visit https://creativecommons.org/licenses/by/4.0/

REVIEWER COMMENTS:

Reviewer #1 (Remarks to the Author):

Review of the manuscript entitled "Amazonian trees under long-term drought exposure are more resilient to El Niño extremes" by Martius et al.

In this paper, the authors show that the eco-physiological resilience and resistance of trees pre-exposed to experimental drought for more than 20 years (PT) are higher than those of untreated controls (CT) in response to the 2023 El Niño drought.

I find this study original and highly relevant. The use of a long-term throughfall exclusion experiment provides a valuable and a rare opportunity to assess how prolonged drought exposure shapes forest resilience and resistance to drought. The analyses are solid, statistically robust, and supported by multiple lines of evidence. Overall, I have no major concerns and support the publication of this work. I do have a few minor comments and suggestions for clarification.

Authors: We would like to thank the reviewer for their positive and very encouraging feedback. Thank you for taking the time to revise our manuscript. We believe that your thoughtful comments have improved our paper. Please find our point-by-point responses to your suggestions below.

General comments

I wonder whether the presence of plastic panels in the throughfall exclusion setup may influence soil evaporation. Could this contribute to preserving soil moisture, or is this effect negligible? A brief discussion would be helpful.

Authors: This is an excellent question. Looking at our soil moisture data in the TFE, we cannot detect evidence of moisture retention due to the panels, particularly as the absolute level of moisture was consistently lower in the TFE. We want to highlight that the absolute soil water content ($m^3\ m^{-3}$) in the TFE was consistently lower. However, due to past mortality, the biomass-relative soil moisture in the TFE was lower during the ENSO enhanced drought. We added Figure 5 as a new figure to the discussion section showing the biomass-relative and absolute soil water content for both plots during the measurement period. With Fig. 5 we have now put the soil moisture plot (soil moisture/biomass) from the Supplementary into the main text. In addition, we added a panel below with the absolute volumetric water content/plot. As you can see, the TFE plot shows consistently lower absolute VWC in the soil compared to the control. However, soil moisture normalised by AGB shows how trees in both plots have similar soil moisture available before the drought, but diverge during the drought. If the panels were to introduce some artificial wetting of the soils, we think we would see it in the soil data, but it doesn't seem to happen and the soils of the TFE are still drier (in absolute terms). We further note that the structure of the roof allows air mixing above and below - it is not 100% sealed. This was evident in temperature records before high mortality. After high mortality there is more turbulence from canopy gaps, so whilst solar radiation increases direct heating in places, increased turbulenceat forest floor mixes the water vapour more fully. The evidence is that the soil is very dry

at the surface, so this would also limit any 'extra' evaporation. Hopefully this has answered your question. Thanks for raising this.

Could vapor pressure deficit (VPD) differ between CT and TFE plots, given the differences in evapotranspiration you report? This potential discrepancy might be worth discussing as, if relevant, it can influence the plant water status stringency calculation.

Author: Thank you for raising this important point. We agree that differences in canopy structure and evapotranspiration between the CT and TFE plots could lead to local differences in VPD, which may influence estimates of plant water status stringency. In structurally heterogeneous tropical forests, microclimatic conditions can vary locally even within plots, as canopy gaps, mortality, leaf shedding/flushing, and local crown exposure all affect atmospheric coupling. This complexity is likely particularly pronounced in the TFE plot, where long-term drought has increased canopy openness and structural heterogeneity. Consequently, some smaller trees may experience substantially higher local VPD due to direct canopy exposure, whereas others remain buffered beneath taller crowns, highlighting the limitations of using a single point measurement to characterise plot-scale atmospheric conditions. In our study, VPD measurements from sensors installed at the top of the towers (~42 m) showed generally comparable temporal dynamics between plots. Hence, we conclude that transpiration dynamics were not driven by larger VPD in one plot. However, detailed vertical VPD profiles within the canopy were unfortunately not available, and the challenge of representing near-crown atmospheric conditions was further compounded by the increasingly open and vertically heterogeneous canopy structure in the TFE treatment. In addition, a logger malfunction in the TFE plot during 2023 reduced the temporal resolution of subcanopy VPD measurements for that period. Hence, we cannot rule out the possibility raised by your review, but added a section on this to the discussion and also acknowledging that local VPD heterogeneity may affect, and potentially underestimate, atmospheric drought stringency in the TFE plot [317-322].

Minor comments

P3, l43: “Amazônia” - portuuese ?

Authors: Thank you for spotting this. We changed it into the English form “Amazonia” [48].

P3, l52: Consider noting that the influence of the NAO on Amazonian drought is relatively minor compared to ENSO.

Authors: Yes, this is a good point, thank you very much. The point you raise is well summarised in the review by Marengo et al (2024): Long-term variability, extremes and changes in temperature and hydrometeorology in the Amazon region: A review. Published In *Acta Amazonica* and I also added the primary source (Cai et al. 2020). I added the sentence that the majority of severe droughts in the Amazon are ENSO related to highlight this, especially in the light of the predicted upcoming El Niño [52-53].

P3, l54: Please cite more recent references. Also, Cai et al. (2014) focuses only on ENSO rather than NAO. My understanding is that the 2005 and 2010 droughts were primarily associated with warmer SSTs in the tropical North Atlantic, shifting the ITCZ northward, rather than NAO influence per se.

Authors: Yes, you are absolutely right. The droughts of 2005 and 2010 cannot be related to NAO per se, but to warmer SST in the TNA (tropical north Atlantic). I updated the paragraph and changed NAO to TNA, following the description/approach by Marengo et al 2024, which was also added as more recent literature + Cai 2020. [49-54].

P12, l294: This sentence is somewhat confusing and appears to contradict your main findings: “Both plots had similar soil-water availability on a tree biomass basis at the start of and following the drought, consistent with eco-hydrological stabilisation.” This seems at odds with your explanation that increased resistance in PT trees arises from reduced competition for soil water following long-term structural adjustments. I’m guessing you are comparing conditions before vs. after the 2023 drought within each treatment, rather than between treatments?

Authors: We thank the reviewer for highlighting this ambiguity and we recognise that the wording was unclear. To improve clarity, we have now added Fig. 5 to the main Discussion, showing both absolute and biomass-normalised soil moisture. Both Fig. 5A and the statistical analyses indicate that biomass-normalised soil water availability (i.e., tree-available soil moisture) was not significantly different between plots prior to the ENSO drought. During the ENSO drought, however, reduced precipitation increased competition for soil water. Because the control plot maintains higher biomass and leaf area, water competition became stronger under drought conditions than in the long-term droughted PT plot, which has undergone structural thinning over time. As shown by Sanchez-Martinez et al. (2025), this long-term adjustment contributes to eco-hydrological stabilisation of the droughted system. Our results further suggest that these structural adjustments also increased resistance to the 2023 ENSO drought, as the lower biomass in the PT plot reduced competition for limited water resources during the dry season.

We have rephrased the sentence accordingly to clarify that the differences in biomass-relative soil moisture availability emerge primarily during ENSO drought conditions rather than under pre-ENSO drought conditions [280-288].

P14, l349: Is species composition similar between plots? It would be nice for the reader to clarify the tree sampling strategy between CT and PT plots. In particular, could differences in species composition (e.g., a higher proportion of drought-tolerant species in PT plots) partly explain the observed differences in resilience? It’s discussed in the method section but it would be useful in the discussion (which is before the method)

Author: Thank you for you comment. We have added a table with species information and with some trait data to the Supplementary Information and have further responded to the comment below and in more detail, as this was also raised by another reviewer. [Supplementary Information Table S5 + line in M&M 428-440]

P18–19 (Soil water content per unit biomass):

Please provide more detail on how soil water content was measured down to 4 m depth. Ensure consistent use of acronyms between the text and equations (e.g., H for height), and avoid including units within equations.

Author: Thank you! This was indeed missing. We updated the M&M section: Soil water content per unit biomass [457-466]

P20:
Would it be more appropriate to estimate soil water potential (ψ_soil) from measured soil water content and soil characteristics, rather than relying on predawn leaf water potential (Ψ_pd)?

Author: This is an excellent question and we have been thinking about this possibility. However, estimating soil water potential (Ψ_soil) from measured soil moisture and soil characteristics introduces an important challenge in tropical forests: the depth from which trees extract water likely differs substantially among individuals and species due to variation in rooting depth and rooting strategies. If we were to assign all trees the same soil layer (e.g., 4 m depth), we could systematically overestimate or underestimate the actual soil water potential experienced by individual trees, depending on whether their active roots occur shallower or deeper than that layer.

In addition, soil water potential can vary strongly with depth during drought, while trees may access water from multiple soil layers simultaneously. Under these conditions, a soil measurement at a single depth may not accurately represent the water available to the plant. We therefore relied on predawn leaf water potential (Ψ_pd), which is widely used in plant hydraulic studies as an integrative proxy for plant-accessible water status, based on the assumption that plant and soil water potentials approximately equilibrate overnight.

We acknowledge that Ψ_pd is not necessarily identical to Ψ_soil, particularly under conditions of nocturnal transpiration, rhizosphere resistance, or incomplete equilibration during drought. Nevertheless, in the absence of detailed soil water potential profiles across the full rooting zone of each tree, we consider Ψ_pd to provide the most physiologically relevant and comparable estimate of water availability across individuals in this study. We added a small sentence to the M&M section [M&M, 503-507].

Reviewer #3 (Remarks to the Author):

Main Comments

I appreciate many aspects of this manuscript. It investigates the resistance and resilience of Amazonian trees under the unprecedent 2023-24 El Niño-driven drought. This is an important contribution, as most studies in the Amazon have focused primarily on drought resistance, whereas the integration of resistance and resilience is essential for ecophysiological understanding and modeling efforts. However, the presented findings raise two important conceptual issues that I encourage the authors to address more explicitly:

Author: We would like to thank the reviewer for their positive feedback and their time and useful comments, that have shaped and enhanced this manuscript substantially.

Paradox #1 -> Primed trees (PT) exhibited lower declines in transpiration during drought progression and higher recovery compared to control trees (CT). The authors suggest that "resilience to extreme events can emerge from long-term drought exposure through structural adjustments at both ecosystem and individual tree scale". This is a strong result and is consistent with empirical practices such as the acclimatization or "hardening" of seedlings before their transfer from nursery environments to field conditions. However, these findings appear to contrast with theoretical frameworks such as alternative stable state theory (ASST). Hammond (2020) (https://doi.org/10.3389/ffgc.2020.560409), for example, suggests that trees surviving prolonged drought may retain hydraulic damage, which can reduce resilience. In that framework, legacy effects of hydraulic failure can prevent full recovery to pre-drought physiological states and increase the probability of ecosystem transition. Similarly, Flores et al. (2024) (https://www.nature.com/articles/s41586-023-06970-0) highlight that ecosystems approaching tipping points often lose resilience even before a state shift occurs. Hysteresis dynamics are also commonly associated with such processes (e.g., Bretfeld et al., 2018 https://doi.org/10.1111/nph.15071; Brum et al., 2026 https://onlinelibrary.wiley.com/doi/full/10.1111/pce.70498 ; Gimenez et al., 2024 https://doi.org/10.1016/j.agrformet.2024.110052).
In this context, I recommend that the authors explicitly discuss how their findings relate to, complement, or potentially challenge these frameworks. In particular, it would be valuable to clarify whether the observed responses represent a form of enhanced resilience through a potential structural reorganization in terms of plant hydraulics, rather than contradicting existing theory.

Author: We thank the reviewer for this thoughtful and important comment. In response, we substantially expanded the Discussion to better position our findings within the context of alternative stable state theory, hydraulic legacy effects, hysteresis dynamics, and recent tipping-point frameworks. We now explicitly clarify that, while our work seems to challenge ASST, we suggest that long-term drought exposure can simultaneously drive structural and physiological reorganisation that modifies subsequent drought responses. Specifically, we discuss how mechanisms such as acclimation, ecohydrological stabilisation, mortality-driven structural adjustment, and altered competitive interactions may buffer surviving trees against future drought extremes despite substantial ecosystem-level costs in biomass and productivity. We further highlight that commonly used resilience proxies, such as critical slowing down, may not fully capture the ecological mechanisms underlying

recovery dynamics. We therefore frame our findings as complementing existing theory by providing mechanistic evidence for how long-term biological and structural adjustments can shape resilience trajectories in Amazon forests [367-384].

Paradox #2 (Role of species composition and functional traits)

The study compares 40 trees (20 per plot) and reports higher resistance and full recovery in PT, versus lower resistance and limited recovery in CT (Figure 1). These differences are attributed primarily to structural traits such as crown size, LAI, and PAVD (Figures 3 and 4).

However, it remains unclear to what extent species composition may contribute to these patterns. The observed patterns emerge from a selected set of individuals, and interspecific variation in functional traits could partially influence the reported differences. For example, if primed trees are dominated by faster-growing, low wood-density species, they may have greater sapwood area and higher internal water storage (capacitance), which could "apparent enhance" both drought resistance and recovery.

I strongly recommend that the authors provide a table (main text or supplementary material) listing the sampled species, along with key attributes such as DBH, wood density, and any available functional traits. This would greatly improve the interpretability of the results.

Additionally, Figure 2 suggests that control trees may reach more negative midday leaf water potentials during peak drought and higher recoveries in predawn leaf water potentials (panel C), which may indicate higher drought tolerance or a more isohydric regulation strategy (see Maréchaux et al., 2015, doi: 10.1111/1365-2435.12452). Furthermore, a brief discussion of stem capacitance and internal water storage would strengthen the physiological interpretation of resistance and resilience.

Authors: We thank the reviewer for this important suggestion. In response, we have added a supplementary table listing all sampled individuals together with key measured traits. The table includes species identity, DBH, wood density, leaf water potential at turgor loss point, and leaf water content at turgor loss point as measures of drought tolerance (Supplementary Table S5). We also updated our statement to the sampling design in the M&M [428-440].

To minimise potential taxonomic and size-related bias, sampling was designed to include individuals from the same species where possible or, where exact matches were not possible, congeneric taxa and similar size classes across plots. Owing to differences in species composition and species availability between treatments, a fully paired design was not achievable, and the resulting dataset therefore represents a partially stratified but unbalanced sampling design. Importantly, we found no significant differences between plots in mean wood density, DBH, or measured leaf hydraulic traits, suggesting that the observed treatment differences are unlikely to be primarily explained by systematic differences in species functional composition. In addition, taxonomic identity was incorporated as a grouping term in our models to account for potential interspecific variation. We have clarified these points in the Supplementary Information and Methods section.

Thank you also for your second comment. I see the issues related to the iso-/anisohydricity and that the lower $\Delta\Psi$ in the TFE might at first suspect that these trees were more stringent, following the concept of iso-/anisohydricity. However, this concept has received substantial criticism (see Hochberg

et al. (2018) https://doi.org/10.1016/j.tplants.2017.11.002) as it is highly dependent on the environment that the plant grows in, and should also be sensitive to environmental exposure, which can be influenced by crown position and size see also Mencuccini et al. (2024) https://doi.org/10.1111/gcb.17222).

However, due to a smaller crown in PTs the water demand is less and this might be reflected in a smaller ΔΨ in the Primed trees. The classical concept of iso-/anisohydricity would interpret this as stronger regulation in the PTs. This is where the stringency metric (Mencuccini et al. (2024)) is better suited in separating the environmental forcing from the tree regulation effect on the Ψ of the tree, as it accounts specifically for the reference well-watered condition. And here is where we see PTs to have smaller g_tot/k_L ratio under well-watered conditions (even under more conservative percentiles (99th, 95th and even 90th – see Figure 4. S4.) – in line with what to expect when the total leaf area/crown size is smaller, which we see in the TFE. Going back to the interpretation of the ΔΨ for the concept of iso-/anisohydricity: This concept is does not separate differences in crown sizes and its interaction with the environment [322-327]. However, in relation to Ψ-md during the drought and its recovery after the drought, these simply seem to follow the pattern of relative soil water availability and the differences during the drought between plots (see Fig. 5).

In addition, we added a small statement to the importance of internally stored water to buffer from drought, in line with a revision request from the second reviewer, that addressed differences in soil moisture threshold. In this context, we also cited a recently published study on threshold responses of soil water content that triggers rapid stem water depletion [294-297].

Specific comments

Methods Section -> Please include a table (main or supplementary) with the list of sampled species list and relevant functional traits (e.g., DBH, wood density, growth rates if available). This will help the readers to better interpret the observed patterns.

Author: This was indeed missing. We included the list of the sampled individuals, as well as some basic trait information, and whether they differ between plots in the Supplementary Information (Table S5), as described in more detail above.

Figure 1 -> A recent study has identified soil moisture thresholds controlling transpiration across Amazon forests. In particular, sap flow responses from multiple species indicate a critical threshold in soil moisture at approximately 0.32 $m^3 m^{-3}$ (at ~0.5 m depth), below which transpiration declines rapidly. (see: Chambers et al., 2025, https://doi.org/10.1038/s41586-025-09728-y). In your study, a similar threshold-like behavior appears to be present in Figure 1, where hydraulic function declines in both treatments, with a slight delay between them. I encourage the authors to explore and discuss this potential threshold response, as it may provide additional mechanistic insight into the observed dynamics.

Author: We thank the reviewer for this important suggestion. We think this has improved the discussion substantially and provided more context. In response, we have expanded the discussion to address the behaviour observed in Figure 1 in the context of recently identified soil moisture thresholds regulating transpiration in Amazonian forests (Chambers et al., 2025). We now discuss how soil moisture in the control plot during the 2023 drought persistently declined below the proposed

critical threshold (~0.32 $m^3$ $m^{-3}$), in line with reductions in transpiration and hydraulic function. We also cite a paper that identifies critical soil moisture thresholds in different ecosystems (> 0.26 m3/m3 in humid, and ~0.12 m3/m3 in arid system), and demonstrates how this shift depending on Leaf Area and aridity (Fu et al., 2024). We discuss this in the light of absolute soil moisture differences between plots. We further relate these dynamics to recent work on critical soil moisture thresholds associated with depletion of internal stem water reserves and discuss how differences in biomass and leaf area between treatments may influence the effective threshold regulating transpiration. These additions are now included in the Discussion section (we added Fig. 5 to the Discussion) [291-301].
