# Supplementary material for: Amazonian trees under long-term drought exposure are more resilient to El Niño extreme
Source: Commun Earth Environ. 2026 Sep 14;7(1):738. doi: 10.1038/s43247-026-03887-0 (PMC13574715; doi:10.1038/s43247-026-03887-0)
Supplement: Supplementary file 2 [file 43247_2026_3887_MOESM2_ESM.pdf]

Supplementary Information

This document comprises the supplementary information for the manuscript entitled:
Amazonian trees under long-term drought exposure are more resilient to El Niño extreme.

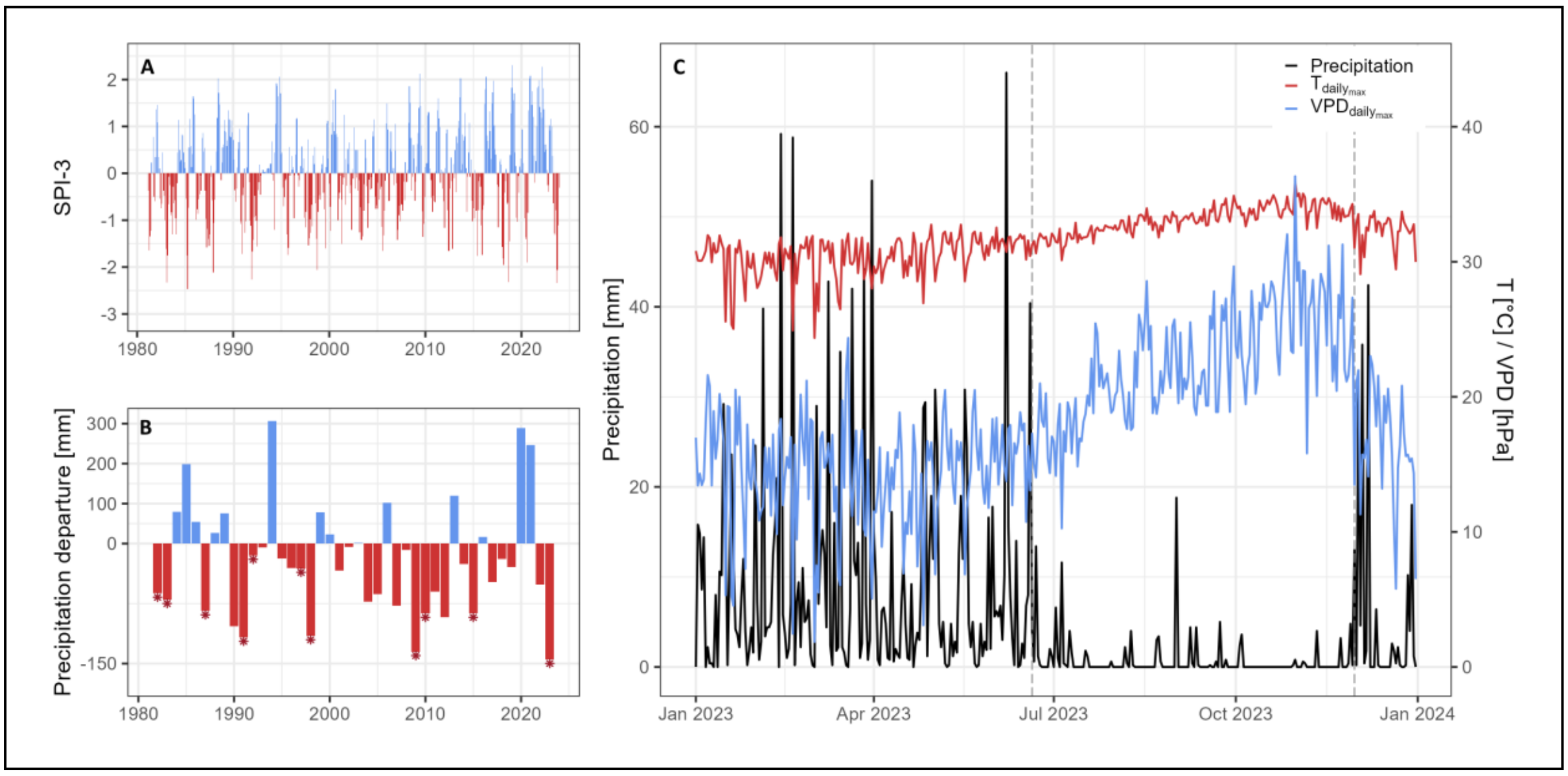

Supplementary Fig. 1: A) Standardised Precipitation Index accumulated over a 3-month period (SPI-3) reflecting short term soil moisture deficits. Months, where SPI ≤ −2.0, are considered as extremely dry, as was the case during our study period in September and October 2023. **B)** Precipitation departure [mm] from long-term dry season mean (Aug-Nov 1982-2022) in Caxiuanã, Amazônia. The dry season of 2023 displayed the lowest rainfall since records began in 1982. El Niño years were marked with an asterisk (*). Note the different y-axis scaling for negative and positive precipitation departure. **C)** The climate of El Niño 2023 as described by daily aggregated precipitation (black), daily maximum temperatures (red) and vapour pressure deficit (blue). Temperature and VPD were measured above the canopy (h = 42 m). Vertical dashed lines delineate the drought period from the 23rd of June – 28th of November 2023.

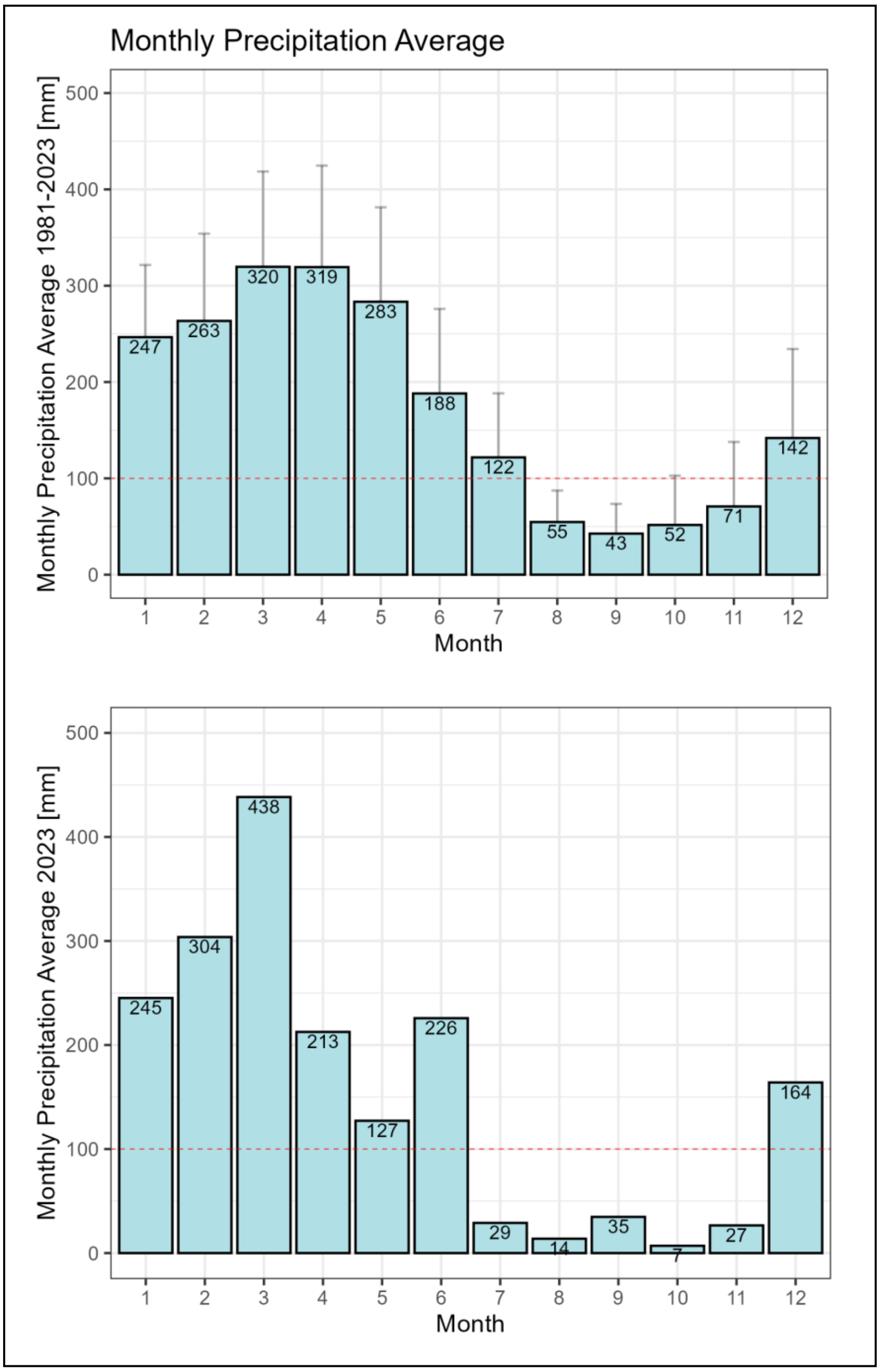

Supplementary Fig. 2: Above) Average monthly precipitation in Caxiuanã (1982-2022). Dry seasons are defined as months where precipitation drops below 100 mm. Hence, the dry season in Caxiuanã is defined as the period between August and November. The effect of the through-fall exclusion experiment was effectively to simulate the recurrence of an El Niño-type soil drought by exacerbating dry season water limitation for >20 consecutive years by increasing the length and intensity of each dry season. During the wet season, however, rainfall remained sufficient—monthly totals exceeded 100 mm even with 50% exclusion—allowing for partial recovery. Thus, trees in the TFE were not continuously droughted for 12 months per year. **Below)** Monthly precipitation during an El Niño year in 2023. The El Niño effectively increases the length of the dry season by 1 month while intensifying it with anomalously low dry-season precipitation.

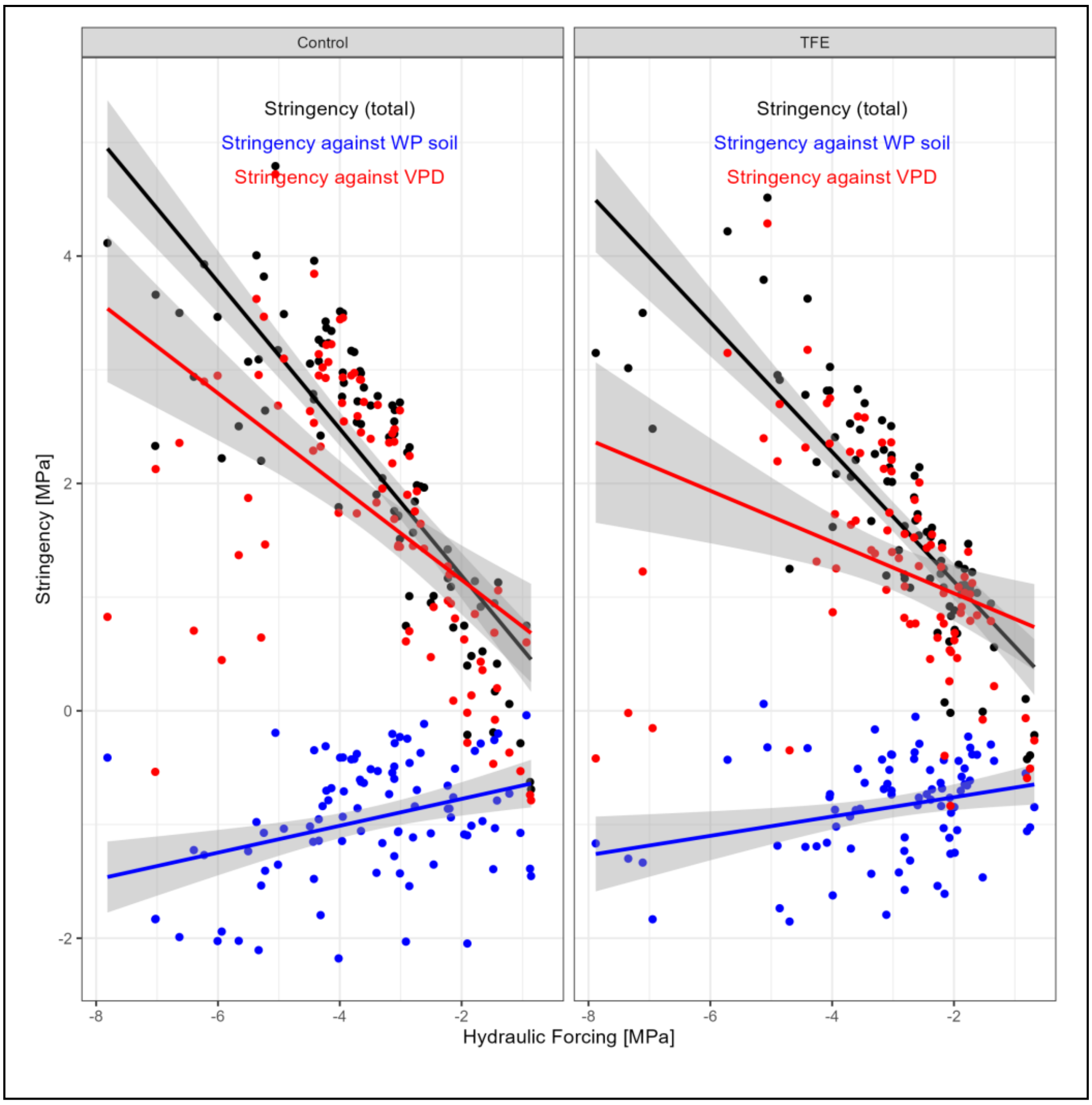

Supplementary Fig. 3: Plant water status stringency regulation aggregated over five campaigns in 2023/24 between plots. The S^{ws} quantifies the departure of observed Ψ_{md} from the predictions of a null model representing both thermodynamic forces (i.e., Ψ_{soil} and VPD) when no physiological regulation occurs. The black line demonstrates the full stringency, and the red and blue lines show the regulation against the VPD and soil WP respectively. Stringency was calculated according to Mencuccini et al. (2024).

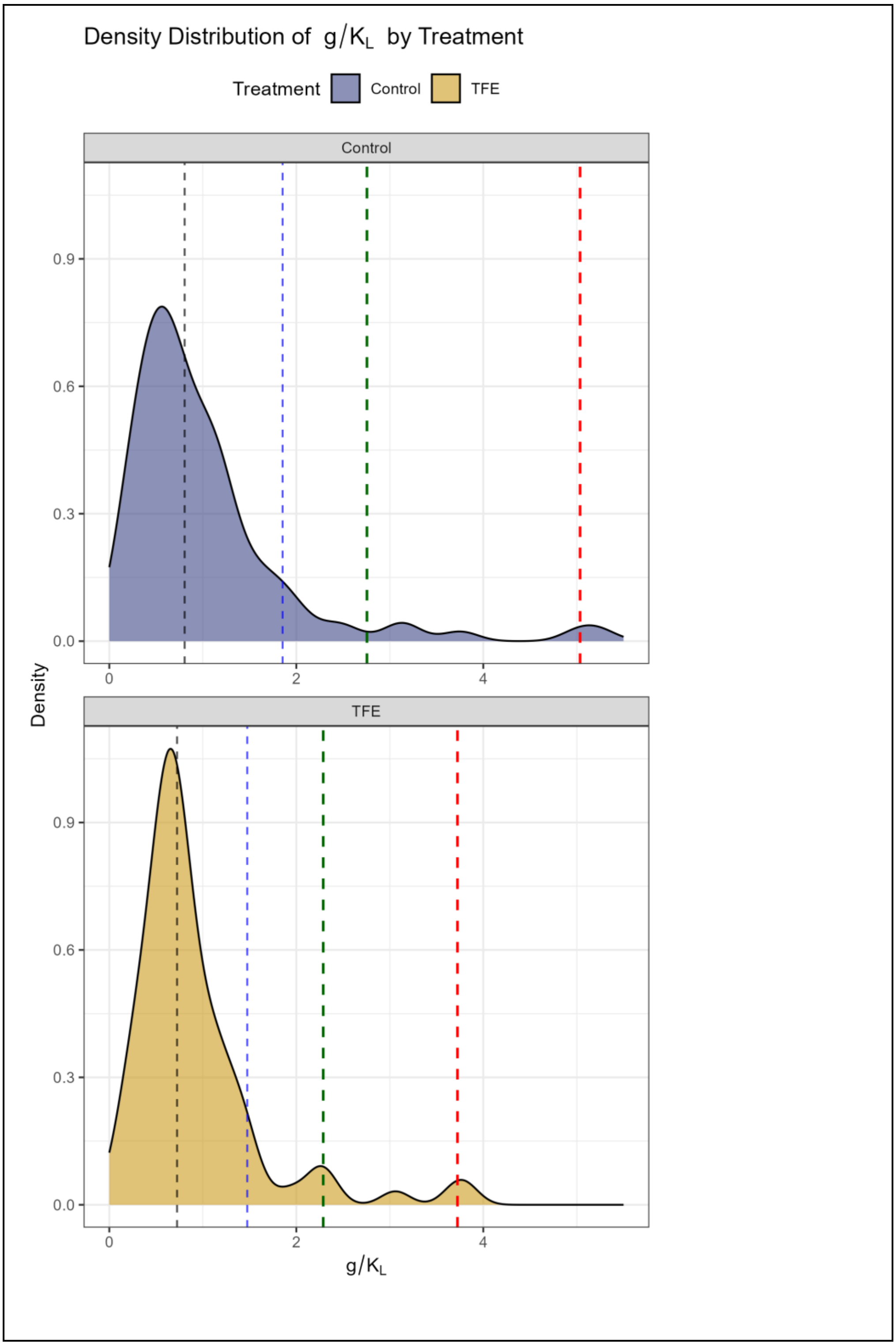

Supplementary Fig. 4: Density distribution of the plot specific ratio of $\frac{g_{tot}}{k_L}$, which indexes the variability of leaf conductance per unit of leaf-specific hydraulic supply capacity on plot level. The dashed lines display the 99th (red), 95th (green) and 90th (blue) percentile of the plot specific distribution. The stringency was calculated on plot level, and thus the more conservative 95th quantile was chosen.

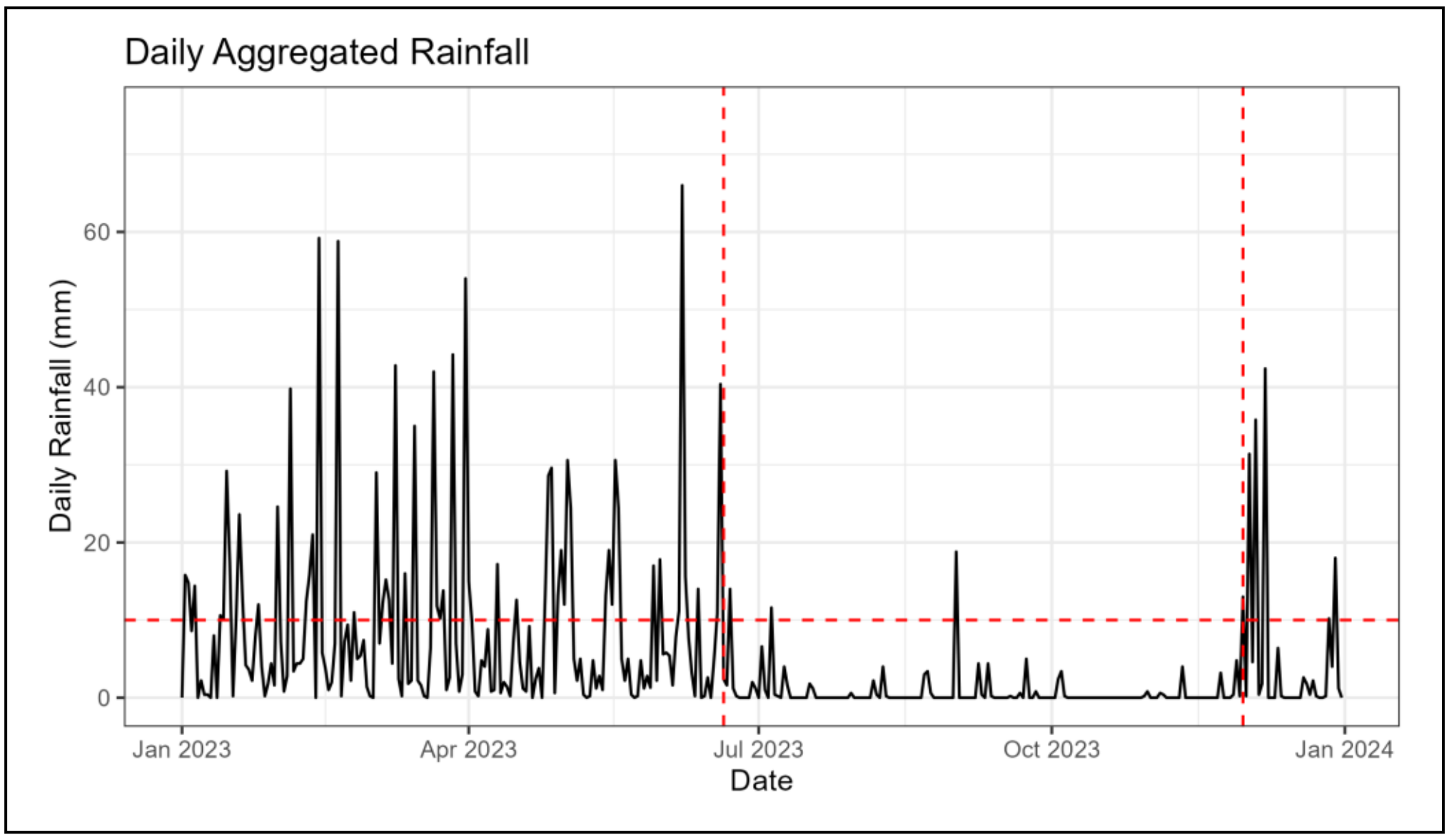

Supplementary Fig. 5: Daily rainfall [mm] in Caxiuanã in 2023. The dry season (Aug-Nov) displayed a water deficit of 145 mm compared to the long-term dry season average. Rainfall dropped significantly from the 22nd of June and stayed below 10 mm day^{-1} (two exceptions) until rainfall resumed on the 27th of November 2023. We thus defined these dates as the drought onset and breaking respectively.

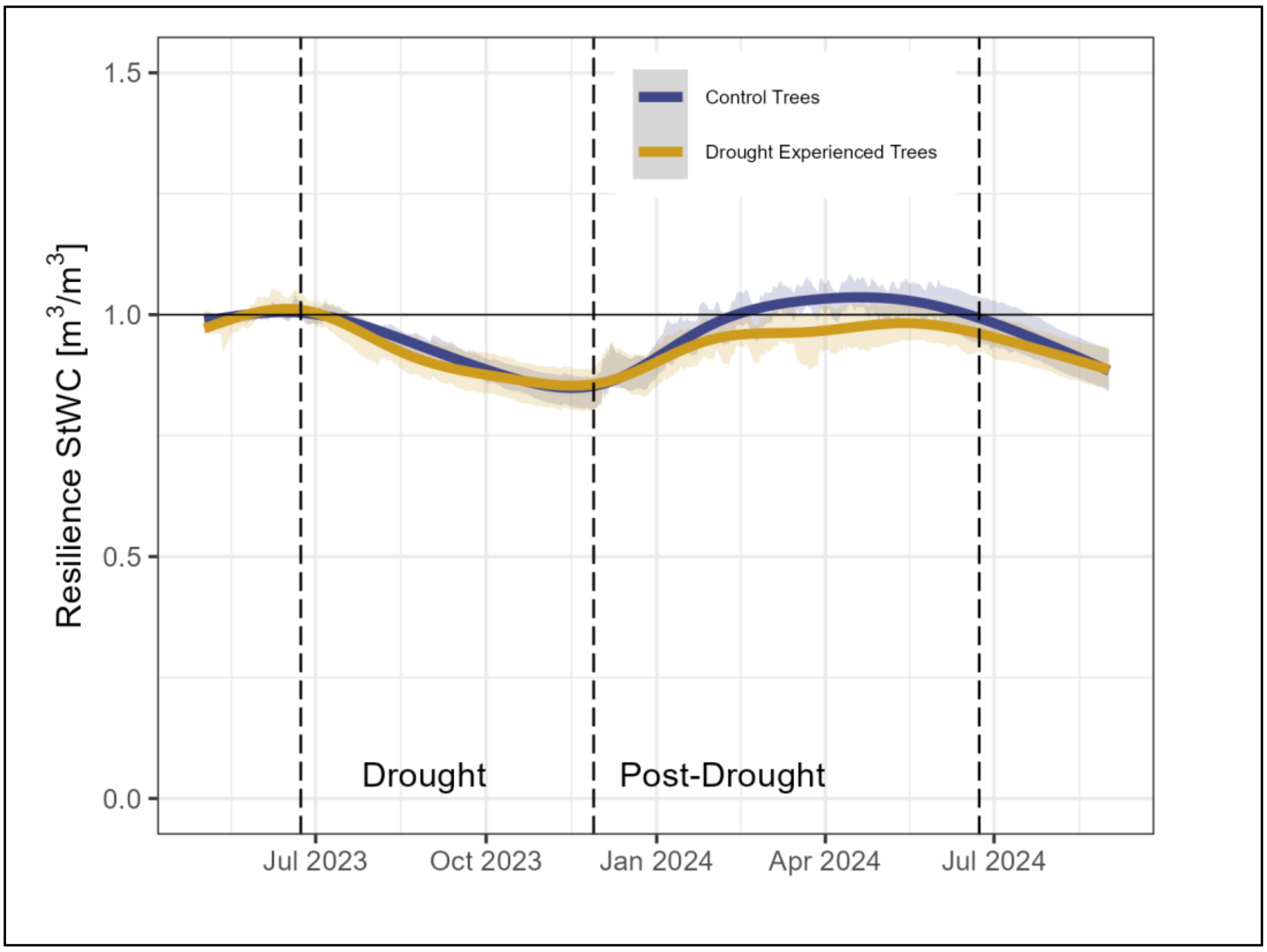

Supplementary Fig. 6: Independent stem water content measurements demonstrate the lack of lag between drought onset and returning rainfall between treatments. Both plots showed similar sensitivity to the defined drought-onset and to returning rainfall. This gives us further support that choosing one drought-onset for both plots. Stem water content was measured using modified Teros12 sensors and calibrated and installed according to Martius et al. (2024).

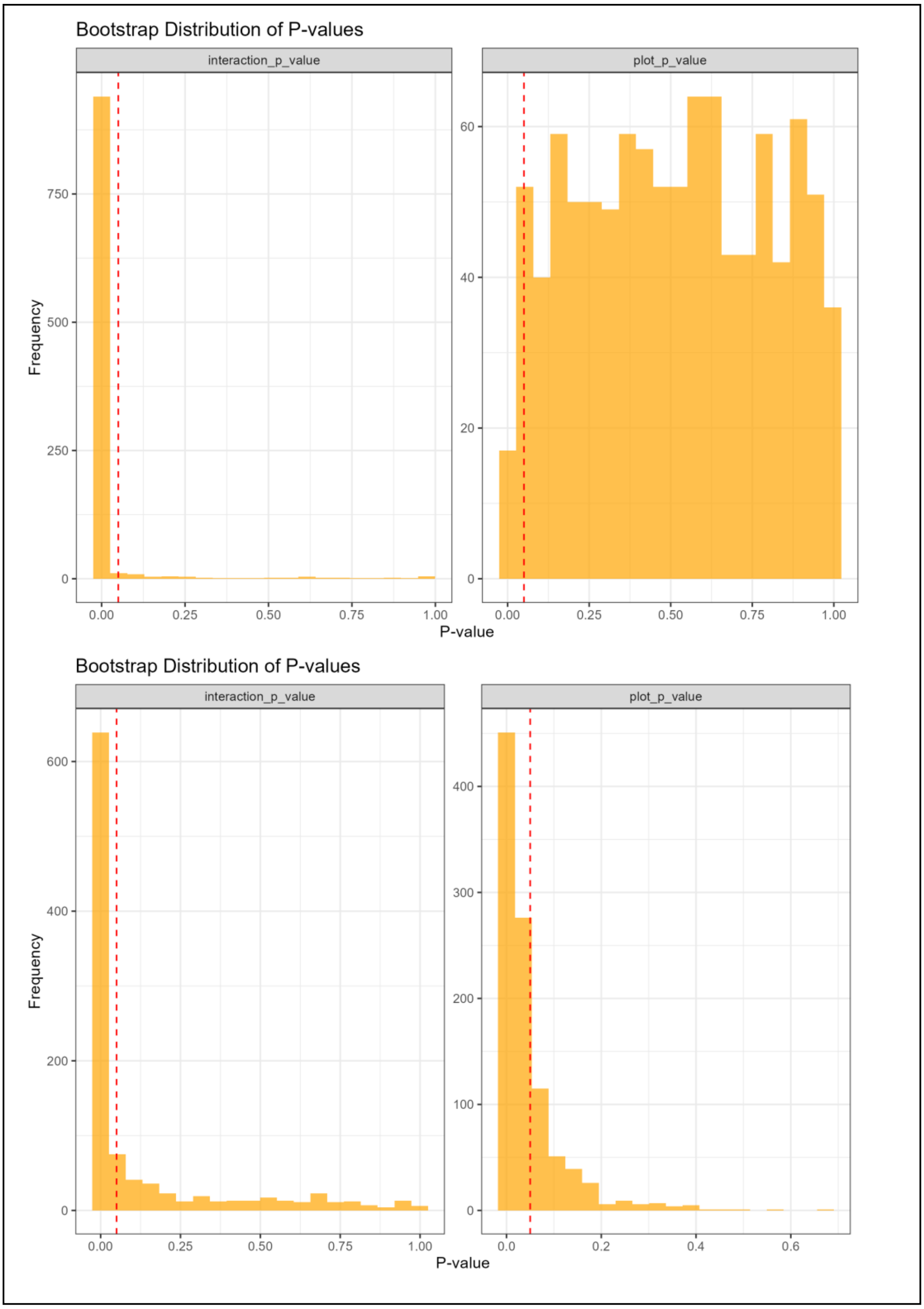

Supplementary Fig. 7: To verify the robustness of our resilience findings despite the relatively low sample size, we performed bootstrap resampling analysis with 1,000 iterations, randomly sampling trees with replacement from each plot and refitting the two linear mixed models (during drought (upper panel) and post-drought (lower panel)) to each bootstrap sample. This approach tested whether the observed treatment effects and temporal interactions remained consistent across different combinations of trees, addressing potential concerns about sample size limitations. All significant effects of the linear mixed model remained significant, giving strong robustness on the sampling design. Plot effects for the resistance model (upper right) was rarely significant, converging with our main model results, meaning that the baseline resistance was not difference between plots at the start of the drought.

Linear Regression Tables

Supplementary Table 1: Regression Table for linear mixed models of drought resistance (Rt) and post-drought resilience (Rs) of transpiration [kg day^{-1}] for Amazonian rainforest trees. The drought period was defined between the 23rd of June – 28th of November 2023, with the post-drought period lasting until 01st of July 2024. This table presents the results from the simple linear mixed model, including only plot differences in transpiration over time. Owing to pre-drought baselining, resilience was standardised to 1.0 at the onset of the drought, with no significant difference between treatments (p = 0.86). As the drought progressed, resilience declined in both treatments, but at a significantly slower rate in TFE trees than in control trees (p < 0.001), indicating greater drought resilience under throughfall exclusion. At the onset of the recovery period, TFE trees exhibited significantly higher resilience than their respective controls (p = 0.007), reflecting their reduced resilience loss during the drought.

Response	Fixed Effects	Estimate	Std. error	DenDf	t-value	p-value
$Rt_{Transpitation}$	Intercept	1.0	0.055	39	18	<2e-16
	Days	-0.0036	0.00010	5137	-35	<2e-16
(28/06 – 28/11/23)	Plot (TFE)	-0.014	0.08	38	-0.17	0.86
	Days x Plot (TFE)	0.0019	0.00014	5137	13	<2e-16
$Rs_{Transpiration}$	Intercept	0.56	0.058	36	9.9	<2e-16
	Days	0.0012	0.00005	6179	21	<2e-16
(29/11/23 -	Plot (TFE)	0.24	0.082	36	2.9	0.007
01/07/24)	Days x Plot (TFE)	-0.0002	0.00007	6182	-3.0	0.002

Supplementary Table 2: Regression Table for linear mixed models of drought resistance (*Rt*) and post-drought resilience *Rs* of transpiration [kg day^{-1}] for Amazonian rainforest trees. The drought period was defined between the 23rd of June – 28th of November 2023, with the post-drought period lasting until 01st of July 2024. This table represents the results of the linear mixed model, including crown size (logarithmic of crown volume) as an additional fixed effect. Numeric fixed effects were scaled for model interpretability. While the effect of crown size had no effect on the absolute resilience of trees (p = 0.72), the rate of resilience loss was larger in trees with larger crowns (p < 0.001).

Response	Fixed Effects	Estimate	Std. error	DenDf	t-value	p-value
$Rt_{Transpitation}$	Intercept	0.70	0.055	35	13	<2e-16
	Days *(scaled)*	-0.0034	0.00011	5037	-33	<2e-16
(28/06 – 28/11/23)	Plot (TFE)	0.23	0.082	35	2.3	0.03
	Crown volume (log-scaled)	0.06	0.04	35	1.4	0.15
	Days x Plot (TFE) (*scaled*)	0.0012	0.00015	5036	9.7	<2e-16
	Days x Crown volume (log-scaled)	-0.0008	0.00007	5034	-11.7	<2e-16
$Rs_{Transpiration}$	Intercept	0.69	0.06	35	11.7	<2e-16
	Days *(scaled)*	0.0011	0.00005	6177	20.2	<2e-16
(29/11/23 -	Plot (TFE)	0.20	0.09	35	2.3	0.03
01/07/24)	Crown volume (log-scaled)	-0.02	0.04	35	-0.36	0.72
	Days x Plot (TFE) (*scaled*)	-0.0002	0.00008	6179	-2.08	0.03
	Days x Crown volume (log-scaled)	0.0001	0.00004	6183	2.7	0.005

Supplementary Table 3: Regression Table for linear mixed models of drought resistance (*Rt*) and post-drought resilience *Rs* of transpiration [kg day^{-1}] for Amazonian rainforest trees. The drought period was defined between the 23rd of June – 28th of November 2023, with the post-drought period lasting until 01st of July 2024. This table represents the results of the linear mixed model, including crown size (logarithmic of crown volume) and soil water content per unit biomass [mm/MgC] as additional fixed effects. Numeric fixed effects were scaled for model interpretability. Biomass-relative water availability explained a significant proportion of the differences in resistance trajectories between plots ($p < 0.001$) and was significantly larger in the TFE compared to the control plot at the peak of the drought ($p < 0.001$).

Response	Fixed Effects	Estimate	Std. error	DenDf	t-value	p-value
$Rt_{Transpitation}$ (28/06 – 28/11/23)	Intercept	0.85	0.06	41	14.5	<2e-16
	Days *(scaled)*	-0.0017	0.0005	5033	-3.06	0.002
	Plot (TFE)	0.08	0.085	40	0.97	0.34
	Crown volume (log-scaled)	0.06	0.04	35	1.46	0.15
	Soil water content [mm/MgC] (*scaled*)	0.2	0.07	5035	2.9	0.003
	Days x Plot (TFE) (*scaled*)	-0.0006	0.0005	5034	-1.37	0.171
	Days x Crown volume (log-scaled)	-0.0008	0.00007	5032	-12.5	<2e-16
	Days x Soil water content [mm/MgC] (*scaled*)	0.0057	0.0006	5036	9.3	<2e-16
$Rs_{Transpiration}$ (29/11/23 - 01/07/24)	Intercept	0.62	0.05	37	10.9	<2e-16
	Days *(scaled)*	0.0004	0.00015	5471	2.4	0.015
	Plot (TFE)	0.30	0.09	37	3.2	0.0025
	Soil water content [mm/MgC] (*scaled*)	0.13	0.02	5469	5.8	<2e-16
	Days x Plot (TFE) (*scaled*)	0.0006	0.00015	5472	4.4	<2e-16
	Days x Soil water content [mm/MgC] (*scaled*)	0.001	0.0003	5470	4.3	<2e-16

Supplementary Table 4: Regression Table for linear mixed models of plant water status stringency during drought, post-drought and plot specific legacy effects tested as differences pre- to post-drought for Amazonian rainforest trees. The drought period was defined between the 23rd of June – 28th of November 2023, with the post-drought period lasting until 01st of July 2024. During the drought period, trees in the TFE plot displayed significantly lower levels of water status regulation ($p = 0.009$). During the post-drought recovery phase, there were no significant differences in the level of water status regulation between plots ($p = 0.68$).

Response	Predictor	Estimate	Std. error	DenDf	t-value	p-value
S^{ws}- Drought (data collection campaigns: 05/23, 07/23, 10/23)	Intercept	1.6	0.16	100	10.2	<2e-16
	Plot (TFE)	-0.6	0.17	44.0	-2.7	0.009
	Campaign Jul-23	0.55	0.17	86	3.0	0.003
	Campaign Oct-23	1.62	0.17	86	9.55	<2e-16
S^{ws} Post-drought (data collection campaigns: 02/24, 05/24)	Intercept	2.0	0.18	76	11	<2e-16
	Plot (TFE)	-0.09	0.21	42	-0.4	0.68
	Campaign (May 24)	-0.9	0.21	42	-4.2	<2e-16

Table S5: Sampled individuals of the study and relevant traits measured for each individual tree. There were no differences between the plot means of wood density (WD, g cm^{-3}), diameter at breast height (DBH), and leaf water potential at turgor loss point (WP- TLP). Differences were assessed using two-sample t-test. The test results and significance levels were stated at the end of the table. Sampling was designed to minimise taxonomic and size-related bias by selecting individuals from comparable species or, where not possible, congeneric taxa and similar size classes across plots. However, due to differences in species composition and availability between plots, exact taxonomic matching was not always achievable. The resulting dataset therefore represents a partially stratified, taxonomically constrained but unbalanced sampling design rather than a fully paired design. Our models incorporate taxonomic identity as a grouping term.

Plot	ID	Species	WD	DBH	WP - TLP
Control	Control_204	*Swartzia racemosa*	0.85	52.84	1.36
Control	Control_211	*Licania octandra*	0.79	29.92	2.20
Control	Control_215	*Pouteria cladantha*	0.79	70.66	1.52
Control	Control_216	*Vouacapoua americana*	0.70	44.25	2.02
Control	Control_218	*Swartzia racemosa*	0.85	29.44	0.67
Control	Control_220	*Goupia glabra*	0.73	67.80	NA
Control	Control_256	*Pouteria decorticans*	0.69	15.60	1.60
Control	Control_259	*Manilkara paraensis*	0.86	77.35	1.76
Control	Control_262	*Eschweilera coriacea*	0.85	40.11	1.70
Control	Control_264	*Micropholis venulosa*	0.67	29.28	0.89
Control	Control_279	*Rinorea guianensis*	0.78	17.19	NA
Control	Control_308	*Pseudopiptadenia suaveolens*	0.68	87.22	2.45
Control	Control_312	*Pouteria cladantha*	0.70	28.65	1.91
Control	Control_313	*Licania octandra*	0.83	22.44	2.07
Control	Control_315	*Pouteria decorticans*	0.69	17.83	2.31
Control	Control_316	*Swartzia racemosa*	0.85	60.16	2.57
Control	Control_317	*Vouacapoua americana*	0.70	20.53	1.26
Control	Control_322	*Vouacapoua americana*	0.70	26.42	1.46
Control	Control_354	*Manilkara bidentata*	0.87	51.41	NA
Control	Control_363	*Pouteria anomala*	0.69	64.62	NA
TFE	TFE_111	*Manilkara bidentata*	0.87	50.93	0.56
TFE	TFE_116	*Pouteria ramiflora*	0.69	24.83	0.50
TFE	TFE_119	*Vouacapoua americana*	0.70	63.98	1.86
TFE	TFE_121.1	*Erisma uncinatum*	0.52	14.96	NA
TFE	TFE_122.1	*Protium pilosissimum*	0.57	11.46	1.91
TFE	TFE_168	*Geissospermum sericeum*	0.78	37.24	1.54
TFE	TFE_169	*Manilkara bidentata*	0.87	58.57	0.95

TFE	TFE_178	*Manilkara bidentata*	0.87	31.51	1.32
TFE	TFE_200	*Protium tenuifolium*	0.57	34.38	1.41
TFE	TFE_207	*Swartzia racemosa*	0.85	47.27	2.27
TFE	TFE_211	*Licania kunthiana*	0.88	29.28	1.91
TFE	TFE_213	*Manilkara huberi*	0.92	22.92	1.63
TFE	TFE_214.1	*Pouteria decorticans*	0.69	10.50	2.51
TFE	TFE_214.3	*Micropholis venulosa*	0.67	13.37	0.71
TFE	TFE_217	*Eschweilera coriacea*	0.85	38.52	1.31
TFE	TFE_266	*Trattinnickia burserifolia*	0.46	159.47	0.85
TFE	TFE_267	*Quararibea guianensis*	0.56	63.18	NA
TFE	TFE_270	*Erisma uncinatum*	0.52	70.98	1.60
TFE	TFE_78	*Hymenolobium flavum*	0.69	25.00	1.62
TFE	TFE_82	*Erisma uncinatum*	0.52	33.42	1.23
TFE		Mean	0.70	42.1	1.43
Control		Mean	0.76	42.7	1.73
		p-value	ns	ns	ns
